# Human histone demethylase KDM6B can catalyse sequential oxidations†
†Electronic supplementary information (ESI) available: Procedures for synthesis of lysine analogues, solid phase peptide synthesis, MS and NMR activity assays. Characterisation of lysine analogues. See DOI: 10.1039/c8cc04057e


**DOI:** 10.1039/c8cc04057e

**Published:** 2018-07-02

**Authors:** Richard J. Hopkinson, Gareth W. Langley, Roman Belle, Louise J. Walport, Kate Dunne, Martin Münzel, Eidarus Salah, Akane Kawamura, Timothy D. W. Claridge, Christopher J. Schofield

**Affiliations:** a Chemistry Research Laboratory , University of Oxford , 12 Mansfield Road , Oxford , OX1 3TA , UK . Email: christopher.schofield@chem.ox.ac.uk; b Leicester Institute of Structural and Chemical Biology and Department of Chemistry , University of Leicester , Lancaster Road , Leicester , LE1 7RH , UK . Email: richard.hopkinson@leicester.ac.uk; c Radcliffe Department of Medicine , Division of Cardiovascular Medicine , BHF Centre of Research Excellence , Wellcome Trust Centre for Human Genetics , Roosevelt Drive , Oxford , OX3 7BN , UK

## Abstract

Jumonji domain-containing demethylases (JmjC-KDMs) catalyse demethylation of *N*^ε^-methylated lysines on histones and play important roles in gene regulation. We report selectivity studies on KDM6B (JMJD3), a disease-relevant JmjC-KDM, using synthetic lysine analogues. The results unexpectedly reveal that KDM6B accepts multiple *N*^ε^-alkylated lysine analogues, forming alcohol, aldehyde and carboxylic acid products.

## Abstract 2

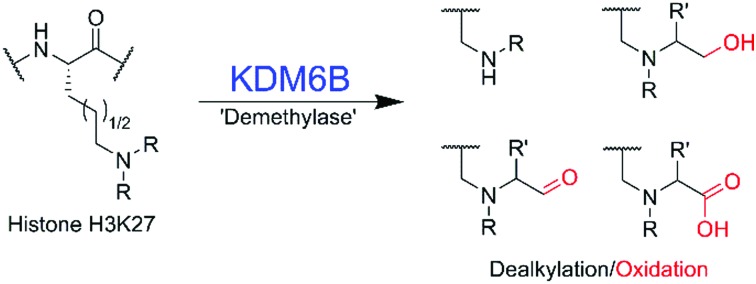
Biochemical studies on the histone lysyl demethylase KDM6B reveal it is capable of catalysing reactions on multiple lysine analogues, forming de-alkylated, hydroxylated, and oxidised products.

## 

The regulation of histone lysyl methylation plays important roles in modulating gene expression and is linked to multiple diseases including cancers and mental disorders.^1^ Jumonji domain-containing protein 3 (KDM6B or JMJD3), a human 2-oxoglutarate (2OG), ferrous iron, and oxygen dependent histone demethylase, removes methyl groups from methylated H3 lysine-27 and is involved in regulating transcription including of genes involved in the production of cytokines (Fig. 1a and b).^2^–^4^ In addition to its action on histones, KDM6B interacts with other proteins such as the tumour suppressor p53,^5^ suggesting KDM6B catalysis may be more promiscuous than presently perceived. Since work with other JmjC-KDMs, and more generally 2OG oxygenases, has revealed the potential for expanded selectivity,^6^–^9^ we undertook substrate profiling studies with KDM6B using synthetic histone fragments incorporating modified lysine analogues. The results reveal that isolated KDM6B accepts a variety of alkylated lysines, resulting in de-alkylated, hydroxylated and carboxylated products. Reactions with an all-carbon analogue of *N*^ε^-trimethyl-lysine did not reveal reaction, implying a positively charged *N*^ε^-alkylamino group is important in KDM6B substrate recognition.

**Fig. 1:**
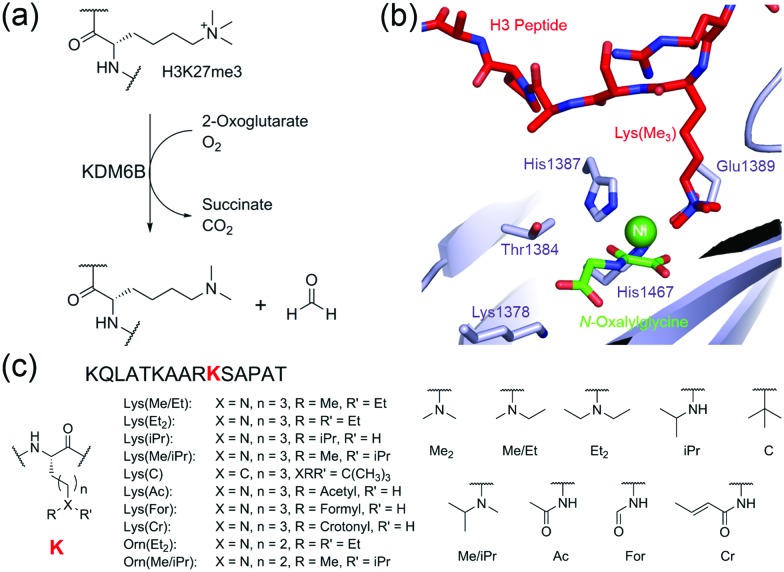
Outline of *N*-demethylation catalysed by KDM6B. (a) Reaction scheme of KDM6B catalysis. Oxidation of an *N*-methyl C–H bond by KDM6B results in formation of an unstable hemiaminal, which fragments to form formaldehyde. (b) View from a crystal structure of KDM6B in complex with histone substrate (PDB ID: ; 4EZH).^12^ The methylated lysine-27 residue (red) protrudes towards the catalytic iron(ii) bound in the active site (for crystallographic purposes, nickel was substituted for iron). *N*-Oxalylglycine (green) is a structural analogue of 2-oxoglutarate. (c) Structures of lysine analogues used in this study. Note: the Lys(Me_2_), Lys(Me/Et), Lys(Et_2_), Lys(iPr) and Lys (Me/iPr) residues are likely protonated under the assay conditions used.

Initially, we synthesised histone H3 peptide fragments containing *N*^ε^-modified lysines and *N*^δ^-modified ornithines at the lysine-27 position (15 residue peptides, sequence KQLATKAAR**K**SAPAT, where **K** corresponds to the analogue, Fig. 1c and Fig. S1, S2, ESI†). All peptides were synthesised as *C*-terminal amides. The fragment peptides were then incubated with KDM6B, 2OG, ascorbate, and ferrous iron (one hour, 25 °C) followed by analysis of the reaction mixtures using MALDI-MS. Clear mass shifts were observed with 5 of the 11 tested peptides, indicating KDM6B catalysed reactions (Fig. 2).

**Fig. 2:**
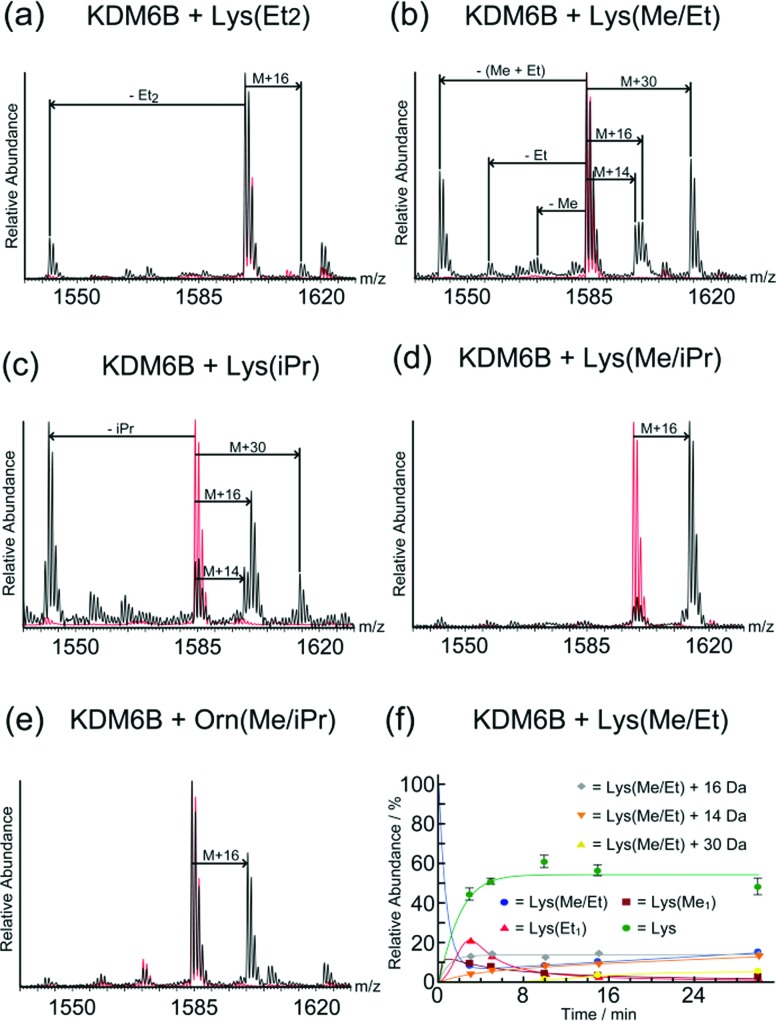
Analysis of KDM6B catalysed oxidations using mass spectrometry. Mass spectra (MALDI) of incubations of KDM6B with (a) KQLATKAAR-Lys(Et_2_)-SAPAT, (b) KQLATKAAR-Lys(Me/Et)-SAPAT, (c) KQLATKAAR-Lys(iPr)-SAPAT, (d) KQLATKAAR-Lys(Me/iPr)-SAPAT, and (e) KQLATKAAR-Orn(Me/iPr)-SAPAT. Mass spectra showing the unmodified peptides are overlaid (red). (f) MS time-course for the incubation of KDM6B with KQLATKAAR-Lys(Me/Et)-SAPAT over 30 minutes. No evidence of KDM6B-catalysed oxidation of the other substrate analogues given in Fig. 1 was observed.

In the samples with the peptide incorporating *N*^ε^-diethyl-lysine (Lys(Et_2_)), a mass shift of –56 Da was observed relative to the starting peptide, implying removal of both ethyl groups (Fig. 2a). A species with a mass 16 Da greater than the starting material was also observed, implying hydroxylation on one of the terminal methyl groups of the diethyl-lysine residue, since hydroxylation at the methylene position leads to de-alkylation.

MS analyses on samples containing KDM6B and *N*^ε^-ethyl-*N*^ε^-methyl-lysine (Lys(Me/Et)) peptide revealed species with masses 14 Da, 28 Da, and 42 Da lower than the starting material, suggesting formation of both *N*^ε^-demethylated and *N*^ε^-de-ethylated products (Fig. 2b). In addition, with the Lys(Me/Et) peptide three other products were observed in the MS experiments, with masses 14 Da, 16 Da and 30 Da heavier than the starting material (Fig. 2b). Time-course studies using MALDI-MS analysis indicated that the Lys(Me/Et) peptide reacts rapidly in the presence of KDM6B, with the fully de-alkylated lysine being the major product over all time points (Fig. 2f). The ‘singly’ demethylated or de-ethylated species were more predominant at early time points, suggesting further de-alkylation of these species occurs to form the fully de-alkylated product. The species with a mass 16 Da heavier than the starting material was observed at all time-points at a constant level; however, the species with masses 14 Da and 30 Da heavier than the starting material increased in concentration during the experiment. The +16 Da, +14 Da and +30 Da mass shifts likely represent sequential oxidation of the methyl group, forming alcohol, aldehyde and carboxylic acid products (as precedented with some other 2OG oxygenases, including the ten-eleven translocation (TET) oxygenases, which are involved in chromatin regulation;^9^–^11^ such sequential oxidation has not been previously reported for JmjC-KDMs, Scheme 1 and Scheme S1, ESI†).

**Scheme 1:**
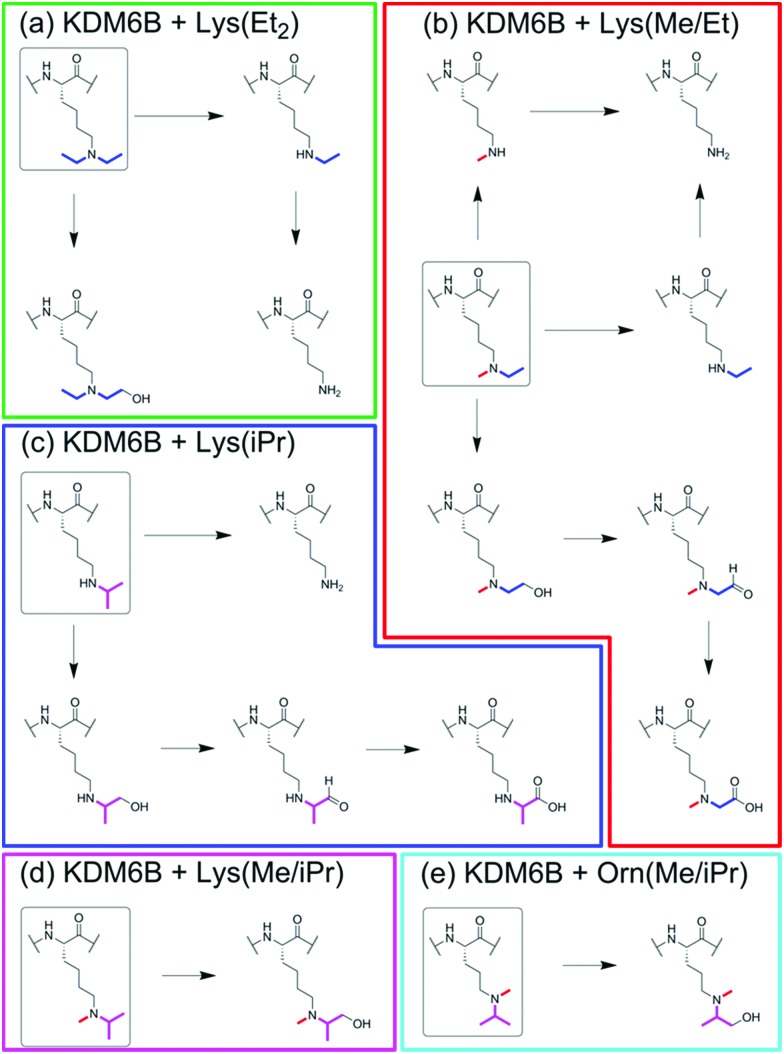
Summarised reactions of KDM6B with histone fragment peptides containing (a) Lys(Et_2_), (b) Lys(Me/Et), (c) Lys(iPr), (d) Lys(Me/iPr), and (e) Orn(Me/iPr), at position 27.

Evidence for reaction was also observed in samples containing the *N*^ε^-isopropyl-lysine (Lys(iPr)) peptide and KDM6B (Fig. 2c). The predominant species observed after incubation possessed a mass 42 Da lower than the starting material, indicating removal of the isopropyl group. ^1^H NMR analyses revealed production of acetone (*δ*_H_ 2.2 ppm), implying KDM6B catalyses oxidation at the sterically hindered tertiary isopropyl C–H bond (Fig. S3, ESI†). As with the Lys(Me/Et) peptide (Fig. 2b) the MS analyses also indicated the formation of products with masses 16 Da, 14 Da and 30 Da heavier than the starting material (Fig. 2c and Fig. S4A, ESI†). These species may be the product of hydroxylation of an isopropyl methyl group, although their relatively low levels in the mixtures precluded their detailed characterisation (note there is potential for stereo-selective methyl group hydroxylation with the isopropyl substrates). Two other peaks were also observed after one hour that possessed masses lower than that of the unmodified peptide, suggesting fragmentation of at least one amino acid side-chain (Fig. S5, ESI†). However, these species were not observed in the time-course analyses (over 30 minutes) and were too low-level for NMR characterisation.

MS analysis of samples with the *N*^ε^-isopropyl-*N*^ε^-methyl-lysine (Lys(Me/iPr)) peptide analogue revealed production of one major product with a mass 16 Da heavier than the starting material (Fig. 2d and Fig. S4B, ESI†). ^1^H NMR analyses reaction revealed low levels of product formation (note: under our NMR conditions, KDM catalysis is lower than observed by MS, possibly as a consequence of the non-catalytically optimised ammonium formate buffer required for NMR and limited oxygen availability in the NMR tube); while the product was present at an insufficient level for full characterisation, comparison of the spectra with those with KDM4E (JMJD2E)^6^ suggested that the product resulted from reaction on an isopropyl methyl group, resulting in a stable alcohol product (as observed with KDM4E, Fig. S6, ESI†).

No reaction was observed between KDM6B and the *N*^δ^-diethyl-ornithine (Orn(Et_2_)) peptide. However, a +16 Da mass shift was observed in samples containing KDM6B and the *N*^δ^-isopropyl-*N*^δ^-methyl-ornithine (Orn(Me/iPr)) peptide (Fig. 2d and Fig. S4C, ESI†). The product of the reaction was tentatively assigned as the Orn(Me/iPr) residue hydroxylated on an isopropyl methyl group, as observed for the Lys(Me/iPr) residue.

Studies were then carried out on the peptide containing the all-carbon analogue (Lys(C)),^7^ Incubation of the Lys(C) peptide with KDM6B at either 25 °C, 37 °C, or with stoichiometric levels of KDM6B did not reveal reaction by MS analysis. Further, ^1^H NMR analyses did not indicate reaction or stimulation of 2-oxoglutarate turnover, which can be observed when demethylases are incubated with substrate analogues.^6^,^7^,^10^ Further, no reaction was observed in samples containing KDM6B and either *N*^ε^-formyl- (Lys(For)), *N*^ε^-acetyl- (Lys(Ac)) or *N*^ε^-crotonyl-lysine (Lys(Cr)) peptides, implying that the positive charge of the side chain amine is important for KDM6B binding/activity (note: no reaction of these residues was observed with either KDM4E or PHF8).^6^,^7^ The lack of oxidation of Lys(For) is interesting given that Lys(Me/Et) and Lys(Me/iPr) residues are likely oxidised to the aldehyde and carboxylic acid derivatives (Scheme 1).

We then profiled the relative activities of the peptides. All analogue-containing substrate peptides (Lys(Et_2_), Lys(Me/Et), Lys(iPr), Lys(Me/iPr) and Orn(Me/iPr) peptides) were poorer substrates than the control (Lys(Me_2_)) peptide, as determined from their relative turnover after one hour (Fig. 2) and time-course studies (Fig. 2f and Fig. S3, S4, S6, ESI† note: the Lys(Et_2_) peptide was too poor a substrate for time-course analysis). The most efficient substrate analogue is the Lys(Me/Et) peptide, followed by the peptides containing Lys(Me/iPr), Lys(iPr), Orn(Me/iPr) and Lys(Et_2_), respectively. The relatively poor activity of the peptides precluded accurate kinetic studies; however, their reduced activity was evidenced by MS competition experiments using a 21-mer Lys(Me_3_) peptide. The results revealed no inhibition of KDM6B-catalysed Lys(Me_3_) demethylation on addition of analogue peptides, within limits of detection, even with a 10-fold excess of the substrate analogue (Fig. S7, ESI†). Further, no evidence for oxidation of the analogue peptides was observed in the same experiments, suggesting the presence of the Lys(Me_3_) peptide is sufficient to out-compete these analogue peptides at the KDM6B active site. No inhibition of KDM6B-catalysed Lys(Me_3_) demethylation was observed upon co-incubation with either the Lys(C), Lys(For), Lys(Ac) or Lys(Cr) peptides (10-fold excess), suggesting they are poor KDM6B binders (Fig. S8, ESI†).

Overall, the results using lysine analogues reveal that KDM6B is capable of accepting multiple modified lysine analogues as substrates and can facilitate oxidation at different positions depending on the structure of the analogue. The observation of hydroxylated products further supports the proposed demethylation mechanism of the 2OG-dependent JmjC demethylases, which is thought to proceed *via* initial *N*-methyl group hydroxylation.^6^–^9^ Our evidence for multiple oxidation events, potentially leading to carboxylated products, supports a close mechanistic relationship between KDM6B and the ferrous iron- and 2OG-dependent TET enzymes (which come from a different structural 2OG oxygenase subfamily compared to the JmjC-KDMs), which sequentially oxidise 5-methyl-cytosine on the 5-methyl group to hydroxymethyl, aldehyde and carboxyl forms in DNA.^11^–^14^ The results also suggest that it may be possible to engineer JmjC-KDM variants that efficiently catalyse reactions other than *N*^ε^-methyl group demethylation, a property that might be useful in dissecting the precise biological roles of these complex multi-domain enzymes. Overall, the available evidence is that the reactivity of KDM6B appears most similar to that of KDM4E, consistent with their structural active site similarity (as revealed by crystallography).^15^,^16^ However, the observed de-ethylation activity of Lys(Et_2_), and the possibility of multiple oxidation reactions occurring on one carbon, reveals that KDM6B can accept bulkier residues into its active site than does KDM4E.^6^ The lack of reaction with the all-carbon analogue of *N*^ε^-trimethyl-lysine implies the importance of the side chain amine for reactivity, an observation that may be useful for the design of peptide-mimetic inhibitors.

We thank the Biotechnology and Biological Sciences Research Council (BB/J003018/1 and BB/L009846/1), Cancer Research UK (C8717/A18245), the Systems Approaches to Biomedical Science Industrial Doctoral Centre (supported by UCB and the Engineering and Physical Sciences Research Council, EP/G037280/1, G. W. L), the People Programme (Marie Curie Actions) of the European Union's Seventh Framework Programme (FP7/2007-2013) under REA grant agreement no. 298603 (Marie Curie IEF Fellowship to M. M), the European Union's Horizon 2020 research and innovation programme under the Marie Skłodowska-Curie grant agreement no. 657292 to L. J. W., and the Wellcome Trust (091857/7/10/7) for funding the work. R. J. H acknowledges a William R. Miller Junior Research Fellowship, St. Edmund Hall, Oxford, UK. K. D. acknowledges support from the BHF Centre of Research Excellence, Oxford (RE/08/004). We also acknowledge a Royal Society Dorothy Hodgkin Fellowship (A. K).

## Conflicts of interest

There are no conflicts to declare.

## Supplementary Material

Supplementary information

## References

[cit1] Pedersen M. T., Helin K. (2010). Trends Cell Biol..

[cit2] Agger K., Cloos P. A., Christensen J., Pasini D., Rose S., Rappsilber J., Issaeva I., Canaani E., Salcini A. E., Helin K. (2007). Nature.

[cit3] De Santa F., Narang V., Yap Z. H., Tusi B. K., Burgold T., Austenaa L., Bucci G., Caganova M., Notarbartolo S., Casola S., Testa G., Sung W. K., Wei C. L., Natoli G. (2009). EMBO J..

[cit4] Yan Q., Sun L., Zhu Z., Wang L., Li S., Ye R. D. (2014). Cell. Signal..

[cit5] Williams K., Christensen J., Rappsilber J., Nielsen A. L., Johansen J. V., Helin K. (2014). PLoS One.

[cit6] Hopkinson R. J., Walport L. J., Münzel M., Rose N. R., Smart T. J., Kawamura A., Claridge T. D. W., Schofield C. J. (2013). Angew. Chem., Int. Ed..

[cit7] Langley G. W., Brinkø A., Münzel M., Walport L. J., Schofield C. J., Hopkinson R. J. (2016). ACS Chem. Biol..

[cit8] Yang M., Hardy A. P., Chowdhury R., Loik N. D., Scotti J. S., McCullagh J. S. O., Claridge T. D. W., McDonough M. A., Ge W., Schofield C. J. (2013). Angew. Chem., Int. Ed..

[cit9] Walport L. J., Hopkinson R. J., Chowdhury R., Schiller R., Ge W., Kawamura A., Schofield C. J. (2016). Nat. Commun..

[cit10] Hancock R. L., Abboud M. I., Smart T. J., Flashman E., Kawamura A., Schofield C. J., Hopkinson R. J. (2018). ChemBioChem.

[cit11] Tahiliani M., Koh K. P., Shen Y., Pastor W. A., Bandukwala H., Brudno Y., Agarwal S., Iyer L. M., Liu D. R., Aravind L., Rao A. (2009). Science.

[cit12] Ito S., Shen L., Dai Q., Wu S. C., Collins L. B., Swenberg J. A., He C., Zhang Y. (2011). Science.

[cit13] Kohli R. H., Zhang Y. (2013). Nature.

[cit14] Cimmino L., Abdel-Wahab O., Levine R. L., Aifantis I. (2011). Cell Stem Cell.

[cit15] Kruidenier L., Chung C. W., Cheng Z., Liddle J., Che K., Joberty G., Bantscheff M., Bountra C., Bridges A., Diallo H., Eberhard D., Hutchinson S., Jones E., Katso R., Leveridge M., Mander P. K., Mosley J., Ramirez-Molina C., Rowland P., Schofield C. J., Sheppard R. J., Smith J. E., Swales C., Tanner R., Thomas P., Tumber A., Drewes G., Oppermann U., Patel D. J., Lee K., Wilson D. M. (2012). Nature.

[cit16] Ng S. S., Kavanagh K. L., McDonough M. A., Butler D., Pilka E. S., Lienard B. M., Bray J. E., Savitsky P., Gileadi C., Von Delft F., Rose N. R., Offer J., Scheinost J. C., Borowski T., Sundstrom M., Schofield C. J., Oppermann U. (2007). Nature.

