# Supplementary material for: Human histone demethylase KDM6B can catalyse sequential oxidations
Source: Chem Commun (Camb). 2018 Jul 2;54(57):7975–8. doi: 10.1039/c8cc04057e (PMC6044289; doi:10.1039/c8cc04057e)
Supplement: Supplementary file 1 [file CC-054-C8CC04057E-s001.pdf]

Electronic Supplementary Material (ESI) for ChemComm.
This journal is © The Royal Society of Chemistry 2018

Supporting Information

Human histone demethylase KDM6B can catalyse sequential oxidations

Richard J. Hopkinson*[a,b], Gareth W. Langley[a], Roman Belle[a], Louise J. Walport[a], Kate Dunne[a,c], Martin Münzel[a], Eidarus Salah[a], Akane Kawamura[a,c], Timothy D. W. Claridge[a], and Christopher J. Schofield*[a]

[a]*Chemistry Research Laboratory, University of Oxford, 12 Mansfield Road, Oxford, U.K., OX1 3TA*
[b]*Leicester Institute of Structural and Chemical Biology and Department of Chemistry, University of Leicester, Lancaster Road, Leicester, U.K., LE1 7RH*
[c]*Radcliffe Department of Medicine, Division of Cardiovascular Medicine, BHF Centre of Research Excellence, Wellcome Trust Centre for Human Genetics, Roosevelt Drive, Oxford, U.K., OX3 7BN*
Email: richard.hopkinson@leicester.ac.uk, christopher.schofield@chem.ox.ac.uk

EXPERIMENTAL PROCEDURES

Materials

N^{α}-Fmoc-N^{ε}-acetyl-lysine and N^{α}-Fmoc-N^{ε}-formyl-lysine were purchased from Bachem. N^{α}-Fmoc-N^{ε}-Boc-N^{ε}-methyl-lysine was from GL Biochem. All other modified lysine analogues were synthesised (see below). Other N^{α}-Fmoc-protected amino acids for solid phase peptide synthesis were purchased from Bachem or AGTC Bioproducts. Buffers, reagents and solvents were purchased from Sigma Aldrich. Reagents were obtained from either Sigma Aldrich, Alfa Aeser, Chem-impex, Bachem, Acros or AGTC Bioproducts. NMR MATCH tubes were from Wilgenburg. Activity assays used KDM6B$_{1141\text{-}1590}$, which was expressed recombinantly in *Escherichia coli*, as previously described[1].

Synthesis of lysine analogues

Synthesis of 2-Fmoc-7-dimethyl-octanoic acid, N^{α}-Fmoc-N^{ε}-crotonyl-lysine, N^{α}-Fmoc-N^{ε}-diethyl-lysine, N^{α}-Fmoc-N^{δ}-diethyl-ornithine, N^{α}-Fmoc-N^{δ}-dimethyl-ornithine, N^{α}-Fmoc-N^{ε}-ethyl-N^{ε}-methyl-lysine, N^{α}-Fmoc-N^{ε}-isopropyl-lysine, N^{α}-Fmoc-N^{ε}-isopropyl-N^{ε}-methyl-lysine and N^{α}-Fmoc-N^{δ}-isopropyl-N^{δ}-methyl-ornithine were synthesised according to previous reports[2, 3]. After synthesis and isolation, the products were used immediately in solid phase peptide synthesis. Synthesis of N^{α}-Fmoc-N^{δ}-isopropyl-N^{δ}-methyl-ornithine hydrochloride is given below. During the work, new procedures for the syntheses of N^{α}-Fmoc-N^{ε}-diethyl-lysine, N^{α}-Fmoc-N^{δ}-dimethyl-ornithine, N^{α}-Fmoc-N^{ε}-ethyl-N^{ε}-methyl-lysine, N^{α}-Fmoc-N^{ε}-diethyl-lysine, N^{α}-Fmoc-N^{ε}-isopropyl-lysine and N^{α}-Fmoc-N^{ε}-isopropyl-N^{ε}-methyl-lysine (all as hydrochlorides) were developed, which provided the compounds in a purified form for characterisation (see below). Reactions were carried out in round-bottomed flasks with magnetic stirring, and were performed under air conditions unless stated otherwise.

For the new syntheses (N^{α}-Fmoc-N^{ε}-diethyl-lysine, N^{α}-Fmoc-N^{δ}-dimethyl-ornithine, N^{α}-Fmoc-N^{ε}-ethyl-N^{ε}-methyl-lysine, N^{α}-Fmoc-N^{ε}-diethyl-lysine, N^{α}-Fmoc-N^{ε}-isopropyl-lysine and N^{α}-Fmoc-N^{ε}-isopropyl-N^{ε}-methyl-lysine), purifications were performed on a Biotage flash purification system with Grace Solve Revelaris or Biotage SiO_2 and Rf-C_{18} columns. The GraceResolve standard settings were as follows: 4 g column (12 mL/min, 1 Column Volume (CV): 5mL), 12 g column (24 mL/min, 16 mL), 24 g column (24 mL/min, 1 CV: 32 mL), 40 g column (40 mL/min, 1 CV: 50 mL), 80 g column (60 mL/min, 1 CV: 100 mL) and 120 g column (80 mL/min, 1 CV: 160 mL). Biotage standard settings were as follows: 10 g column (36 mL/ min, 1 CV: 15 mL), 25 g column (75 mL/min, 1 CV: 33 mL), 50 g column (100 mL/min, 1 CV: 66 mL) and 100 g column (100 mL/min, CV: 132 mL). Fraction collection was at 254 nm and monitoring was at 230 nm. Yields depicted are of isolated material. Reaction monitoring was carried out on Merck thin layer chromatography (TLC) plates with visualisation at 254 nm and by $KMnO_4$ or ninhydrin staining. Monitoring by liquid chromatography-mass spectrometry (LC-MS) was also used. ^{1}H NMR analyses were performed at 400 MHz or 500 MHz. ^{13}C NMR was performed at 100 MHz or 125 MHz on Bruker electrospin 400 with samplXpress or Bruker electrospin 500 spectrometers. Chemical shifts are given in (δ) ppm and couplings are reported in Hz. Abbreviations for couplings are as follows: s = singlet, d = doublet, t = triplet, q = quadruplet, quint = quintet, hept = heptet, b = broad, m = multiplet and app t = apparent triplet. Solvents used for NMR were $CDCl_3$-d_1, MeOD-d_4 or DMSO-d_6, which were all from Sigma Aldrich. Assigned protons are indicated as underlined and italic and aromatic protons on the Fmoc group are assigned as depicted below. Infrared spectra were obtained using an IR Bruker tensor 27 spectrometer and optical rotation data were furnished using a Schmidt Haensch polarimeter with a 10 cm cell at 298 K. Low resolution mass spectrometry was carried out on an Agilent 6120 quadruple LC/MS Agilent 1260 in combination with Waters sample manager 2777. LC-MS analyses were performed using an Agilent 6120 quadruple LC/MS Agilent 1200. All small molecules were analysed in positive ion mode using electron spray ionisation (ESI). Lyophilisation was performed using a Scanvac Coolsafe lyophiliser.

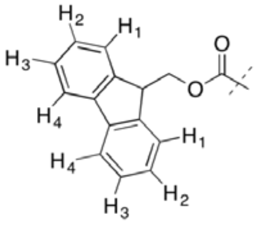

^{1}H NMR Assignment of aromatic Fmoc protons.

(S)-2-[Fluorenylmethoxycarbonyl]amino-5-[isopropyl, methyl]amino-pentanoic acid hydrochloric acid (N^{α}-Fmoc-N^{δ}-isopropyl-N^{δ}-methyl-ornithine hydrochloride)

Fmoc-Orn-OH·HCl (356 mg, 0.91 mmol, 1.0 equiv.) was dissolved in MeOH (10 mL) before addition of acetone (1 mL). $NaCNBH_3$ (86 mg, 1.37 mmol, 1.5 equiv.) was then added and the reaction was left stirring for 2 h. Formalin (0.5 mL, 37%$_{(aq)}$, 6.15 mmol, 7 equiv.) was then added before addition of $NaCNBH_3$ (86 mg, 1.37 mmol, 1.5 equiv.). The reaction was left stirring overnight (12 h) before addition of a few drops of conc. HCl and removal of the volatiles *in vacuo*. Acetone (10 mL) was then added to the residue followed by filtration. The supernatant was then collected and the volatiles were removed *in vacuo*. The resultant white solid (267 mg, 66%) was used directly in sold phase peptide synthesis without purification. mp: 41 °C (decomp.); IR υ_{max}: 1722, 1604, 1405 cm^{-1} $[\alpha]^{25}_D$ +21.0 (c = 0.010 g mL^{-1} MeOH);

^{1}H NMR 500 MHz (DMSO-d$_6$) δ (ppm): 7.90 (d *J* = 7.5 Hz, 2H, 2x FmocC$_4$*H*), 7.72 (app t, *J* = 7.5, 2H, 2x FmocC$_1$*H*), 7.44 (app t, *J* = 7.5 Hz, 2H, 2x FmocC$_3$*H*), 7.33 (app t, *J* = 7.5 Hz, 2H, 2x FmocC$_2$*H*), 4.20-4.33 (m, 3H, OC*H*$_2$C*H*), 3.79-3.83 (m, 1H, $^{\alpha}$C*H*), 3.23-3.31 (m, 1H, NHC(CH$_3$*H*CH$_3$)) 2.66-2.79 (m, 1H, $^{\delta}$CH$_2$), 2.40 (s, 3H, NHC*H*$_3$(CH$_3$CHCH$_3$)), 1.40-1.72 (m, 4H, $^{\beta}$C*H*$_2$$^{\gamma}$C*H*$_2$), 1.06-1.14 (m, 3H, NHC(CH$_3$HC*H*$_3$); ^{13}C NMR 100 MHz (DMSO-d$_6$) δ (ppm): 174.6, 155.9, 128.1, 127.5, 125.6, 65.9, 54.9, 54.6, 52.8, 47.1, 34.0, 29.9, 21.1, 16.5; ESI-MS in MeOH [M+H]$^+$, calc: 411.2 found: 411.2; HRMS (ESI+) in MeOH [M+H]$^+$ as $C_{24}H_{31}N_2O_4^+$, [M+H]$^+$: calc: 411.2278 found: 411.2274.

(S)-2-[Fluorenylmethyloxycarbonyl]amino-6-[diethyl]amino-hexanoic acid hydrochloric acid (*N*$^{\alpha}$-Fmoc-*N*$^{\varepsilon}$-diethyl-lysine hydrochloride)

To a suspension of Fmoc-lys(HCOOH)-OH (801 mg, 1.93 mmol, 1.0 equiv.) in EtOH (20 mL, 0.1M), acetaldehyde (1 mL, 17.8 mmol, 9.23 equiv.) was added followed by slow addition of $NaBH_3CN$ (268 mg, 4.25 mmol, 2.2 equiv.). The mixture was stirred for 45 min creating overtime a clear solution. TFA (300 µL) was added drop wise and the mixture was stirred for 3 min. *In vacuo* volatiles were removed and the crude was loaded on a Grace 40 g R_f-C_{18} column followed by biotage mediated purification. 3 CV (5%) equilibration, loading (~6 mL, 25%), 2 CV (5%), 20 CV (5 to 100%) MeCN in H_2O (containing 0.01% TFA). Selected fractions were lyophilised yielding, 564 mg of white solid (1.33 mmol, 69%). The solid was redissolved in CH_2Cl_2 (5 mL, 0.27 M) and to the solution was added HCl in Et_2O (2 mL, 2 M, 4 mmol, 3.0 equiv.) and volatiles were removed *in vacuo*. Azeotropically Et_2O was removed with CH_2Cl_2 *in vacuo* (4x 10 mL). mp: 76 °C (decomp.); IR υ_{max}: 3407, 2978, 2362, 1695, 1200, 1130 cm^{-1}; $[\alpha]^{25}_D$ +6.5 (c = 0.010 g mL^{-1} MeOH); ^{1}H NMR 400 MHz (MeOD-d$_4$) δ (ppm): 7.65 (d, *J*= 7.5 Hz, 2H, 2×FmocC$_4$*H*), 7.53 (apparent t, *J*= 8.0 Hz, 2H, 2×FmocC$_1$*H*), 7.26 (t, *J*= 7.5 Hz, 2H, 2×FmocC$_3$*H*), 7.18 (t, *J*= 8.0 Hz, 2H, 2×FmocC$_2$*H*), 4.27-4.15 (m, 2H, OC*H*$_2$CH), 4.07 (t, *J*= 7.0 Hz, 1H, OCH$_2$C*H*), 4.00-3.94 (m, 1H, NH$^{\alpha}$C*H*COOH), 3.01 (q, *J*= 7.5 Hz, 4H, CH$_2$$^{\varepsilon}$N(C*H*$_2CH_3$)$_2$), 2.94-2.83 (m, 2H, $^{\delta}$CH$_2$$^{\varepsilon}$C*H*$_2$N), 1.83-1.70 (m, 1H, $^{\alpha}$CH$^{\beta}$CH*H*$^{\gamma}$CH$_2$), 1.66-1.44 (m, 3H, $^{\alpha}$CH$^{\beta}$C*H*H$^{\gamma}$CH$_2$$^{\delta}$C*H*$_2$$^{\varepsilon}CH_2$), 1.37-1.26 (m, 2H, $^{\beta}$CH$_2$$^{\gamma}$C*H*$_2$$^{\delta}CH_2$), 1.12 (t, *J*= 7.0 Hz, 6H, N(CH$_2$C*H*$_3$)$_2$); ^{13}C NMR 100 MHz (MeOD-d$_4$) δ (ppm): 177.8, 158.3, 145.2, 142.5, 128.8, 128.2, 126.2, 120.9, 67.8, 56.2, 52.7, 48.4, 48.1, 32.9, 24.3, 23.8, 9.0; ESI-MS in MeOH [M+H]$^+$, calc: 425.2 found: 425.2; HRMS (ESI+) in MeOH [M+H]$^+$ as $C_{25}H_{33}N_2O_4^+$, [M+H]$^+$: calc: 425.2435 found: 425.2421.

(S)-2-(9-fluorenylmethyloxycarbonyl)amino-5-(dimethyl)amino-pentanioc acid hydrochloric acid (*N*$^{\alpha}$-Fmoc-*N*$^{\delta}$-dimethyl-ornithine hydrochloride)

A mixture of Fmoc-Orn-OH·HCl (990 mg, 2.53 mmol, 1.0 equiv.) in ethanol (20 mL) was stirred for 15 min to a clear solution. HCHO (1.00 mL, 37 %$_{(aq)}$, 13.42 mmol, 2.53 equiv) was added drop wise followed nu the addition of $NaBH_3CN$ (350 mg, 5.57 mmol, 2.20 equiv.). The mixture was stirred for 45 min until ESI-MS indicated reaction completion. To the solution 300 µL of TFA was added drop wise followed stirring for 15 min. The mixture was filtered through some cotton in a funnel, washed with 3x 10 mL of EtOH. The crude was obtained after removal of solvents *in vacuo*. The mixture was dissolved in 8 mL of MeCN/ H_2O (1:5) and was purified on R_f-C_{18} column SNAP cartridge KP-C18-HS. (Gradient program: 2 CV 5% to 5%, 20 CV 5% to 100% of MeCN:H_2O containing 0.01% TFA (v/v%)). Volatiles where removed upon lyophilization obtaining a white fluffy solid of 590 mg (61 %). The combined fractions were dissolved in DCM (5 mL) followed by the addition of HCl in Et_2O (5 mL, 10 mmol, 2 M) to protonate the zwitter ionic specie. Volatiles were removed *in vacuo* obtaining a hydroscopic white solid. mp: 73.4 °C (decomp.); IR υ_{max}: 2955, 2362, 1714, 1534, 1448, 1252,

1082 cm^{-1}; $[\alpha]^{25}_D$ +4.0 (c = 0.010 g mL^{-1} MeOH); ^1H NMR 400 MHz (DMSO-d_6) δ (ppm): 12.77 (s, 1H, $NH^{\alpha}CHCOO\underline{H}$), 10.40 (s, 1H, $^{\delta}CH_2N\underline{H}(CH_3)_2$), 7.92 (d, *J*= 7.5 Hz, 2H, 2×$FmocC_4\underline{H}$), 7.78-7.71 (m, 3H, 2×$FmocC_1\underline{H}$, $N\underline{H}^{\alpha}CHCOOH$), 7.44 (t, *J*= 7.5 Hz, 2H, 2×$FmocC_3\underline{H}$), 7.35 (t, *J*= 7.5 Hz, 2H, 2×$FmocC_2\underline{H}$), 4.37-4.20 (m, 3H, $OC\underline{H}_2CH$, $OCH_2C\underline{H}$), 4.04-3.95 (m, 1H, $NH^{\alpha}C\underline{H}COOH$), 3.09-2.96 (m, 2H, $^{\gamma}CH_2{}^{\delta}C\underline{H}_2NH(CH_3)_2$), 2.72 (d, *J*= 4.8 Hz, 6H, $N(C\underline{H}_3)_2$), 1.81-1.58 (m, 4H, $^{\alpha}CH^{\beta}C\underline{H}_2{}^{\gamma}CH_2$, $^{\beta}CH_2{}^{\gamma}C\underline{H}_2{}^{\delta}CH_2$). ^{13}C NMR (DMSO, 126 MHz) δ 173.4, 156.2, 143.8, 140.7, 127.7, 127.1, 125.3, 120.2, 65.7, 56.0, 53.4, 46.6, 42.1, 41.8, 27.8, 20.7. ESI-MS in MeOH, $[M+H]^+$, found: 383.2, calc.: 383.2. HR-MS in MeOH, $[M+H]^+$, found: 383.1953 calc.: 383.1965.

(*S*)-2-[Fluorenylmethyloxycarbonyl]amino-6-[methyl, ethyl]amino-hexanoic acid hydrochloric acid (*N*$^{\alpha}$-Fmoc-*N*$^{\varepsilon}$-ethyl-*N*$^{\varepsilon}$-methyl-lysine hydrochloride)

To a solution of Fmoc-Lys(me, Boc)-OH (573 mg, 1.19 mmol, 1.0 equiv.) in CH_2Cl_2 (5 mL, 0.24 M) was added HCl in Et_2O (2 mL, 2 M, 4 mmol, 3.3 equiv.) while stirring at RT. The mixture was continued stirring for 16 h followed by the removal of volatiles under reduced pressure obtaining the crude as white solid. To a solution of Fmoc-Lys(me, HCl)-OH in EtOH (15 mL, 0.08 M), acetaldehyde (404 µL, 7.2 mmol, 6.0 equiv.) was added drop wise followed by $NaBH_3CN$ (169 mg, 2.69 mmol, 2.3 equiv.). The mixture was stirred for 1.5 h and progress of the reaction was determined by ESI-MS. TFA (380 µL, 4.57 mmol, 3.84 equiv.) and 1 mL of H_2O was added followed by stirring at RT for 15 min. Volatiles were removed *in vacuo* and the crude was loaded on a Grace 40 g R_f-C_{18} column for biotage purification. 3 CV (5%) equilibration, loading (~15 mL, 25%), 2 CV (5%), 20 CV (5 to 100%) MeCN in H_2O (containing 0.01% TFA). Selected fractions were lyophilized (yielding, 326 mg, 0.79 mmol, 67%), combined and dissolved in CH_2Cl_2 (5 mL, 0.16 M). To the solution was added slowly HCl in Et_2O (1 mL, 2 M, 2 mmol, 1.68 equiv.) while stirring and volatiles were removed *in vacuo*. Azeotropically Et_2O was removed with CH_2Cl_2 *in vacuo* (4x 10 mL) obtaining the title compound as a white solid. mp: 74 °C (decomp.); IR υ_{max}: 3392, 3061, 2944, 1708, 1533, 1201 cm^{-1}; $[\alpha]^{25}_D$ -1.3 (c = 0.011 g mL^{-1} MeOH); ^1H NMR 400 MHz (DMSO-d_6) δ (ppm): 12.67 (s, 1H, $^{\alpha}CHCOO\underline{H}$), 10.25 (s, 1H, $^{\varepsilon}N\underline{H}(CH_2CH_3)CH_3$), 7.90 (d, *J*= 7.5 Hz, 2H, Fmoc-$C_4\underline{H}$), 7.76-7.71 (m, 2H, 2×$FmocC_1\underline{H}$), 7.68 (d, *J*= 8.0 Hz, 1H, $N\underline{H}^{\alpha}CHCOOH$), 7.42 (t, *J*= 7.5 Hz, 2H, 2×$FmocC_3\underline{H}$), 7.34 (t, *J*= 7.5 Hz, 2H, 2×$FmocC_2\underline{H}$), 4.34-4.26 (m, 2H, $OC\underline{H}_2CH$), 4.26-4.18 (m, 1H, $OCH_2C\underline{H}$), 3.94 (dt, *J*= 9.0 4.5 Hz, 1H, $NH^{\alpha}C\underline{H}COOH$), 3.18-3.07 (m, 1H, $^{\delta}CH_2{}^{\varepsilon}CH\underline{H}NH$), 3.06-2.96 (m, 2H, $NH(C\underline{H}_2CH_3)CH_3$), 2.96-2.84 (m, 1H, $^{\delta}CH_2{}^{\varepsilon}C\underline{H}HNH$), 2.67 (d, *J*= 5.0 Hz, 3H, $NH(CH_2CH_3)C\underline{H}_3$), 1.80-1.55 (m, 4H, $^{\alpha}CH^{\beta}C\underline{H}_2{}^{\gamma}CH_2{}^{\delta}C\underline{H}_2{}^{\varepsilon}CH_2$) 1.43-1.27 (m, 2H, $^{\beta}CH_2{}^{\gamma}C\underline{H}_2{}^{\delta}CH_2$), 1.20 (t, *J*= 7.0 Hz, 3H, $NH(CH_2C\underline{H}_3)CH_3$); ^{13}C NMR 100 MHz (MeOD-d_4) δ (ppm): 175.5, 158.7, 145.1, 142.5, 128.8, 128.2, 126.2, 120.9, 67.9, 56.4, 54.9, 52.3, 48.3, 39.7, 32.0, 24.6, 24.0, 9.6; ESI-MS in MeOH $[M+H]^+$, calc: 411.2 found: 411.2; HR-MS (ESI+) in MeOH as $C_{24}H_{31}N_2O_4^+$, calc: 411.2278 found: 411.2261.

(S)-2-[Fluorenylmethyloxycarbonyl]amino-6-[isopropyl]amino-hexanoic acid hydrochloric acid (*N*$^{\alpha}$-Fmoc-*N*$^{\varepsilon}$-isopropyl-lysine hydrochloride)

To a suspension of Fmoc-Lys(HCOOH)-OH (1.012 g, 2.5 mmol, 1.0 equiv.) in ethanol (20 mL) acetone (1 mL, 13.6 mmol, 5.4 equiv.) was added followed by the addition of $NaBH_3CN$ (179 mg, 2.85 mmol, 1.14 equiv). The mixture was monitored until completion with ESI-MS followed by quenching after 45 min with the addition of TFA (300 µL). The mixture was stirred for 3 minutes followed by filtration and washed with 3× of ethanol (10 mL). The supernatant was concentrated *in vacuo* followed by HPLC purification on Rf-C_{18} column. Biotage cartridge KP-C_{18}-HS 30 g R_f-C_{18}, 3 CV 5% equilibration, 2 CV 5%, 20 CV 5% to 100 % of MeCN

(containing 0.01% TFA) in H_2O (containing 0.01% TFA). The selected fractions were collected and combined followed by removal of volatiles by lyophilization. The white compound obtained (727 mg, yielding 71%) was protonated with HCl · Et_2O (5 mL, 2 M, 10 mmol, 4.0 equiv.) followed by the removal of diethyl ether under reduced pressure and finally to the mixture was added 2× CH_2Cl_2 (10 mL) followed by azeotropic removal *in vacuo* of excessive amounts of diethyl ether, obtaining a white solid. mp: 98 °C (decomp.); IR υ_{max}: 3400, 2954, 2362,1703, 1528, 1253, 1050 cm^{-1}; $[\alpha]^{25}_{D}$ -3.3 (c = 0.014 g mL^{-1} MeOH); ^1H NMR 600 MHz (DMSO-d_6) δ (ppm): 12.63 (s, 1H, $NH^{\alpha}CHCOO\underline{H}$), 8.82 (s, 1H, $^{\varepsilon}CH_2N\underline{H}_2(CH(CH_3)))$, 7.90 (d, *J*= 8.0 Hz, 2H, 2×$FmocC_4\underline{H}$), 7.73 (t, *J*= 7.0 Hz, 2H, 2×$FmocC_1\underline{H}$), 7.66 (d, *J*= 8.0 Hz, 1H, $N\underline{H}^{\alpha}CHCOOH$), 7.42 (t, *J*= 8.0 Hz, 2H, 2×$FmocC_3\underline{H}$), 7.33 (t, *J*= 8.0 Hz, 2H, 2×$FmocC_2\underline{H}$), 4.33-4.25 (m, 2H, $OC\underline{H}_2CH$), 4.22 (t, *J*= 7.0 Hz, 1H, $OCH_2C\underline{H}$), 3.94 (dt, *J*= 8.5 3.0 Hz, 1H, $NH^{\alpha}C\underline{H}COOH$), 3.22 (hept, *J*= 6.5 Hz, 1H, $^{\varepsilon}CH_2NH_2(CH_3C\underline{H}CH_3))$, 2.87-2.76 (m, 2H, $^{\delta}CH_2{}^{\varepsilon}C\underline{H}_2NH_2(CH_3CHCH_3))$, 1.77-1.69 (m, 1H, $^{\alpha}CH^{\beta}C\underline{H}H^{\gamma}CH_2$), 1.68-1.55 (m, 3H,$^{\alpha}CH^{\beta}CH\underline{H}^{\gamma}CH_2{}^{\delta}C\underline{H}_2{}^{\varepsilon}CH_2$), 1.45-1.32 (m, 2H, $^{\beta}CH_2{}^{\gamma}C\underline{H}_2{}^{\delta}CH_2$), 1.23 (d, *J*= 6.5 Hz, 6H, $^{\varepsilon}CH_2NH(C\underline{H}_3CHC\underline{H}_3))$; ^{13}C NMR 100 MHz (MeOD-d_4) δ (ppm): 173.8, 156.2, 143.8, 140.7, 127.6, 127.1, 125.3, 120.1, 65.6, 53.6, 49.2, 46.6, 43.5, 30.2, 25.2, 22.8, 18.5; ESI-MS in MeOH, $[M+H]^+$ calc: 411.2, found: 411.3; HR-MS (ESI+) in MeOH as $C_{24}H_{31}N_2O_4^+$, $[M+H]^+$: calc: 411.2278, found: 411.2265.

(S)-2-[Fluorenylmethyloxycarbonyl]amino-6-[methyl, isopropyl]amino-hexanoic acid hydrochloric acid (*N*$^{\alpha}$-Fmoc-*N*$^{\varepsilon}$-isopropyl-*N*$^{\varepsilon}$-methyl-lysine hydrochloride)

To a suspension of Fmoc-Lys(HCOOH)-OH (1.003 g, 2.5 mmol, 1.0 equiv.) in ethanol (20 mL) acetone (1 mL, 13.6 mmol, 5.4 equiv.) was added drop wise followed by the addition of $NaBH_3CN$ (172 mg, 2.7 mmol, 1.08 equiv). The mixture was monitored until completion with ESI-MS followed by the addition of formaldehyde (900 µL, 15.0 mmol, 6.0 equiv.) and $NaBH_3CN$ (169 mg, 2.7 mmol, 1.08 equiv.). The reaction mixture was stirred until completion monitored by ESI-MS and after 1 h the addition of TFA (300 µL) quenched the reaction mixture. The mixture was filtered and the residue was washed 3× with ethanol (10 mL). The supernatant was concentrated *in vacuo* followed by purification on R_f-C_{18} column with biotage. Biotage cartridge KP-C18-HS 30 g R_f-C_{18}, 3 CV 5% equilibration, 2 CV 5%, 20 CV 5% to 100% of MeCN (containing 0.01% TFA) in H_2O (containing 0.01% TFA). The selected fraction were collected and combined followed by removal of volatiles by lyophilization. The white compound obtained (871 mg, yielding 82%) was protonated with HCl · Et_2O (2 mL, 2 M, 4 mmol, 1.6 equiv.) followed by the removal of diethyl ether under reduced pressure and finally the mixture was 2× CH_2Cl_2 (10 mL) was added to remove excessive amounts of diethyl ether obtaining a white solid. mp: 78 °C (decomp); IR υ_{max}: 3400, 2976, 2362, 1709, 1709, 1532, 1251, 1201 cm^{-1}; $[\alpha]^{25}_{D}$ -0.6 (c = 0.020 g mL^{-1} MeOH); ^1H NMR 400 mHz (DMSO-d_6) δ (ppm): 12.67 (s, 1H, $NH^{\alpha}CHCOO\underline{H}$), 9.84 (s, 1H, $^{\varepsilon}CH_2N\underline{H}CH_3(CH_3CHCH_3))$, 7.92 (d *J*= 8.0 Hz, 2H, 2x $FmocC_4\underline{H}$), 7.78-7.72 (m, 2H, 2x $FmocC_1\underline{H}$), 7.69 (d, *J*= 8.0 Hz, 1H, $N\underline{H}^{\alpha}CHCOOH$), 7.44 (t, *J*= 7.5 Hz, 2H, 2x $FmocC_3\underline{H}$), 7.35 (t, *J*= 7.5 Hz, 2H, 2x $FmocC_2\underline{H}$), 4.36-4.20 (m, 3H, $OC\underline{H}_2C\underline{H}$), 3.95 (m, 1H, $NH^{\alpha}C\underline{H}COOH$), 3.56-3.45 (m, 1H, $NHC(CH_3\underline{H}CH_3))$ 3.10-2.98 (m, 1H, $^{\delta}CH_2{}^{\varepsilon}C\underline{H}HNH$), 2.97-2.84 (m, 1H, $^{\delta}CH_2{}^{\varepsilon}CH\underline{H}NH$), 2.62 (d, *J*= 5.0 Hz, 3H, $NHC\underline{H}_3(CH_3CHCH_3))$, 1.82-1.60 (m, 4H, $^{\alpha}CH^{\beta}C\underline{H}_2{}^{\gamma}CH_2{}^{\delta}C\underline{H}_2{}^{\varepsilon}CH_2$), 1.45-1.31 (m, 2H, $^{\beta}CH_2{}^{\gamma}C\underline{H}_2{}^{\delta}CH_2$), 1.25 (d, *J*= 6.0 Hz, 3H, $NHCH_3(C\underline{H}_3CHCH_3))$, 1.20 (dd, *J*= 2.0, 7.0 Hz, 3H, $NHC(CH_3HC\underline{H}_3))$; ^{13}C NMR 100 mHz (DMSO-d_6) δ (ppm): 173.8, 156.2, 143.8, 140.7, 127.7, 127.1, 125.3, 120.1, 65.6, 55.9, 53.6, 51.8, 46.6, 34.6, 30.2, 23.4, 22.9, 16.7, 15.1; ESI-MS in MeOH, $[M+H]^+$ calc: 425.2 found: 425.3; HR-MS (ESI+) in MeOH as $C_{25}H_{33}N_2O_4^+$, $[M+H]^{+:}$ calc: 425.2435, found: 425.2422.

Solid Phase Peptide Synthesis

Peptides were synthesised using a C336X peptide synthesiser (CS Bio, U. S. A.) on MBHA resin using standard Fmoc-based peptide synthesis. The peptides were cleaved from the resin using trifluoroacetic acid / triisopropylsilane (97.5/2.5 % v/v) and purified by reverse-phase high-performance liquid chromatography using a Vydac C18 column (Grace, U. S. A.).

MALDI-MS Activity Assays

MALDI-MS activity assays were carried out on samples containing KDM6B (2 µM), histone fragment peptide (10 µM), 2-oxoglutarate (50 µM), ascorbate (100 µM) and ammonium ferrous sulphate (10 µM, diluted from a 100 µM stock in H_2O) in 50 mM HEPES buffer pH 7.5 (total volume = 20 µL). After incubation for one hour at 25 °C, the samples were quenched with MeOH (40 µL); 1 µL samples were spotted onto plates for analysis using a Waters MALDI Micro MX mass spectrometer. α-Cyano-4-hydroxycinnamic acid (CHCA) was used as the matrix. Observed masses for the peptides were within 1 Da of their predicted mono-charged masses. For time-course experiments, aliquots from the reaction mixtures were taken at different time points before immediate quenching with methanol and spotting onto MALDI plates. Concentrations for each species were determined using the following equation.

$$[Peptide] = \frac{peptide\ signal\ sntensity}{sum\ of\ all\ peptide\ signal\ intensities} \times initial\ substrate\ concentration$$

MALDI-MS Inhibition/Competition Assays

KDM6B (1 µM) was pre-incubated with the histone fragment peptides (100 µM final concentration, 10 minutes, 25°C) prior to initiating reaction by addition of 2-oxoglutarate (50 µM), ascorbate (100 µM), ammonium ferrous sulphate (10 µM, diluted from a 100 µM stock in H_2O) and H3K27Me3 21mer peptide (sequence: KAPRKQLATKAAR-Lys(Me_3)-SAPATGG, 10 µM) in HEPES buffer (50 mM pH 7.5) (total volume = 20 µL). After incubation (3 minutes at 25 °C), reactions were quenched by addition MeOH (40 µL), after which 1 µL of each sample was spotted onto a MALDI plate with α-cyano-4-hydroxycinnamic acid (CHCA) matrix, and then analysed using a Waters MALDI Micro MX mass spectrometer. Percentage methylation and percentage demethylation relative to enzyme free control were calculated as above. Data were analysed using Dunnett's multiple comparison test on GraphPad Prism 7.

NMR Analyses

^{1}H NMR experiments with the Lys(iPr)- and Lys(Me/iPr)-containing peptides and KDM6B were carried out on a Bruker Avance AVIII 700 MHz spectrometer equipped with an inverse TCI cryoprobe optimised for ^{1}H observation and installed with TOPSPIN 2 software. Samples were prepared in Eppendorf tubes before being transferred to 2 mm MATCH NMR tubes and inserted into the spectrometer for analysis. Samples were prepared as follows; KDM6B (2 mg/mL, diluted from a 10 mg/mL stock in 10 mM HEPES, 300 mM NaCl, 1 mM dithiothreitol, 5 % glycerol pH 7.5), histone fragment peptide (100 µM, diluted from a 1 mM stock in H_2O), 2-oxoglutarate (1 mM), ascorbate (100 µM) and ammonium ferrous sulphate (10 µM, diluted from a 100 µM stock in H_2O) in dAFN buffer (total volume = 75 µL, for preparation of dAFN buffer, see reference 4)[4]. The sample with Lys(iPr) also contained (3-(trimethylsilyl)-2,2,3,3-

tetradeuteropropionic acid (0.13 mg/mL) as an internal reference. After a brief optimisation of the spectrometer conditions, time-course data were collected using automated routines; multiple ^{1}H NMR experiments were collected sequentially, with each experiment consisting of 32 transients, with a total acquisition time of 168 seconds. The total lag time between sample preparation and the first analysis was 300-500 seconds. The solvent deuterium signal was used as an internal lock signal and the water resonance was reduced using presaturation or gradient suppression. Experiments with the Lys(Me/iPr) peptide sample were conducted at 292 K (experiments with the Lys(iPr) peptide sample were at 298 K). ^{1}H chemical shifts are reported relative to the solvent resonance (δ_H 4.7 ppm).

SUPPORTING FIGURES

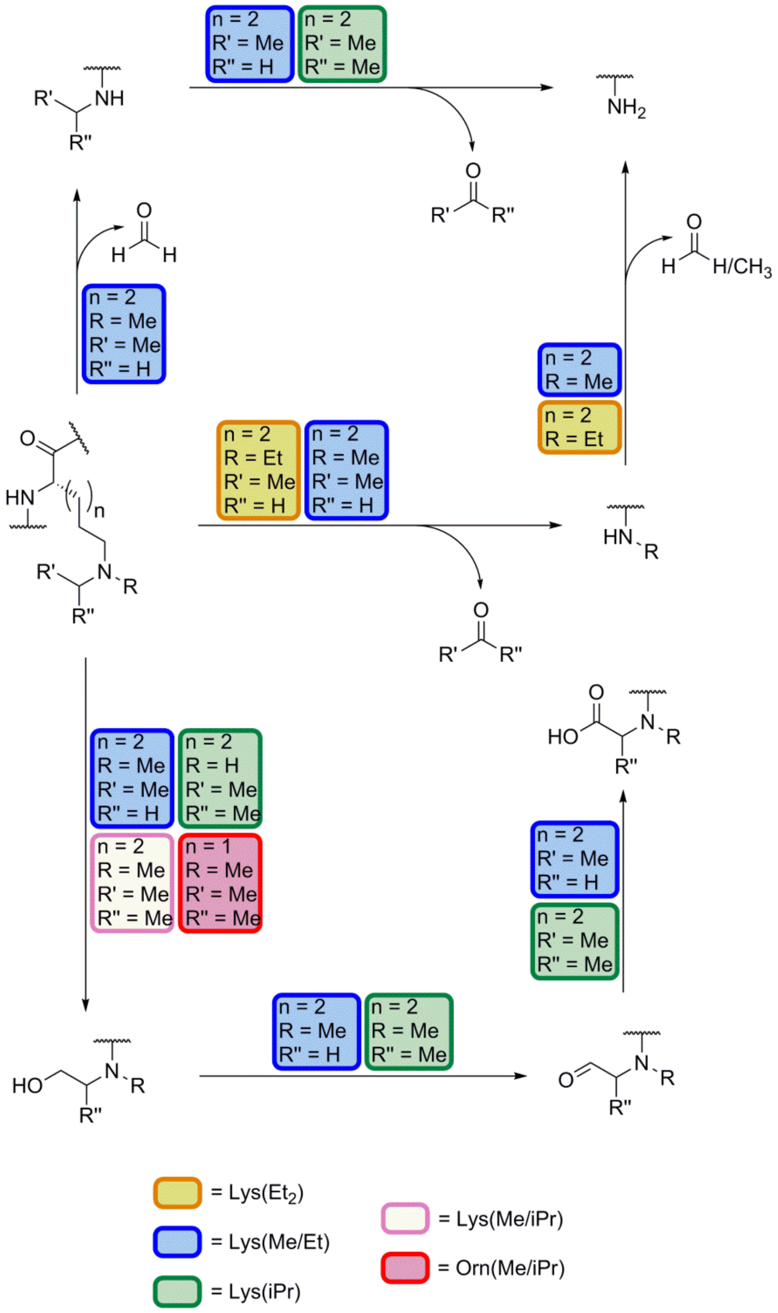

Scheme S1. Schematic representation of the reactions on the Lys(Me/Et), Lys (Et_2), Lys(iPr), Lys(Me/iPr) and Orn(Me/iPr) residues. Reactions with each residue (or intermediate) are colour coded.

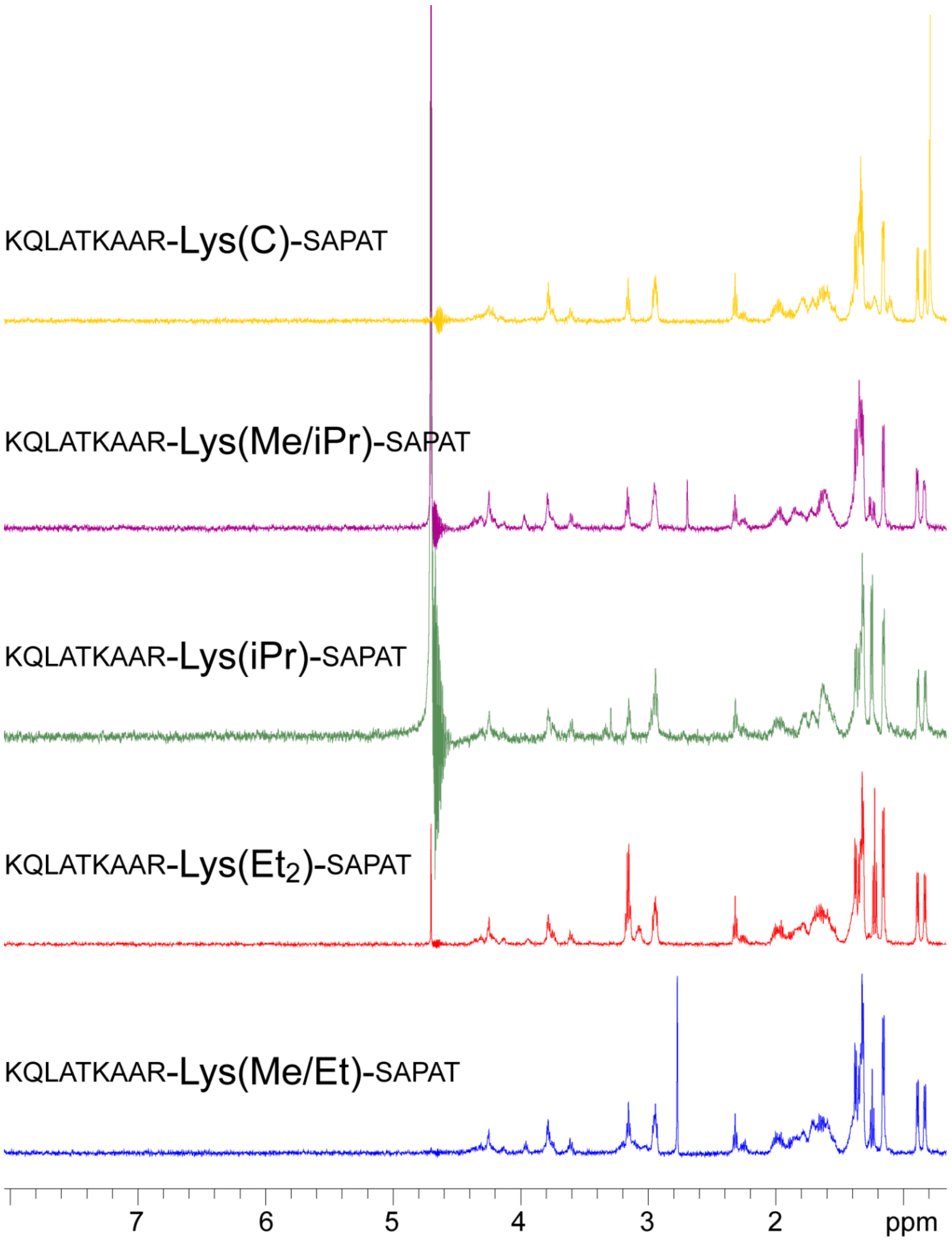

Figure S1. ^{1}H NMR spectra of the lys(Me/Et)-, Lys(Et_2)-, Lys(iPr)-, Lys(Me/iPr)- and Lys(C)-containing histone fragment peptides used in this study. The amino acid sequences for each peptide are given beside the corresponding spectrum.

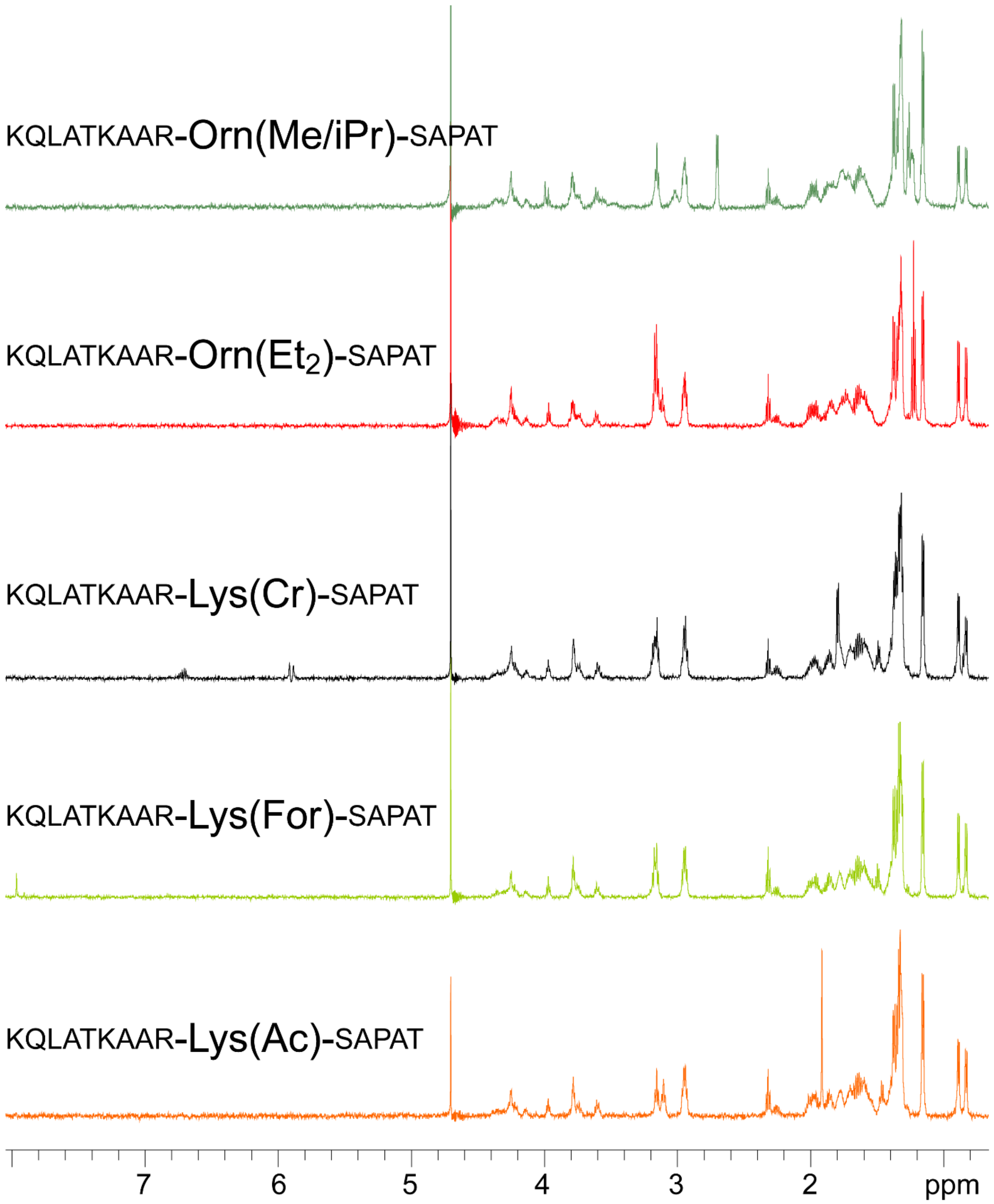

Figure S2. ^{1}H NMR spectra of the Lys(Ac)-, Lys(For)-, Lys(Cr)-, Orn(Et$_2$)- and Orn(Me/iPr)-containing histone fragment peptides used in this study. The amino acid sequences for each peptide are given beside the corresponding spectrum.

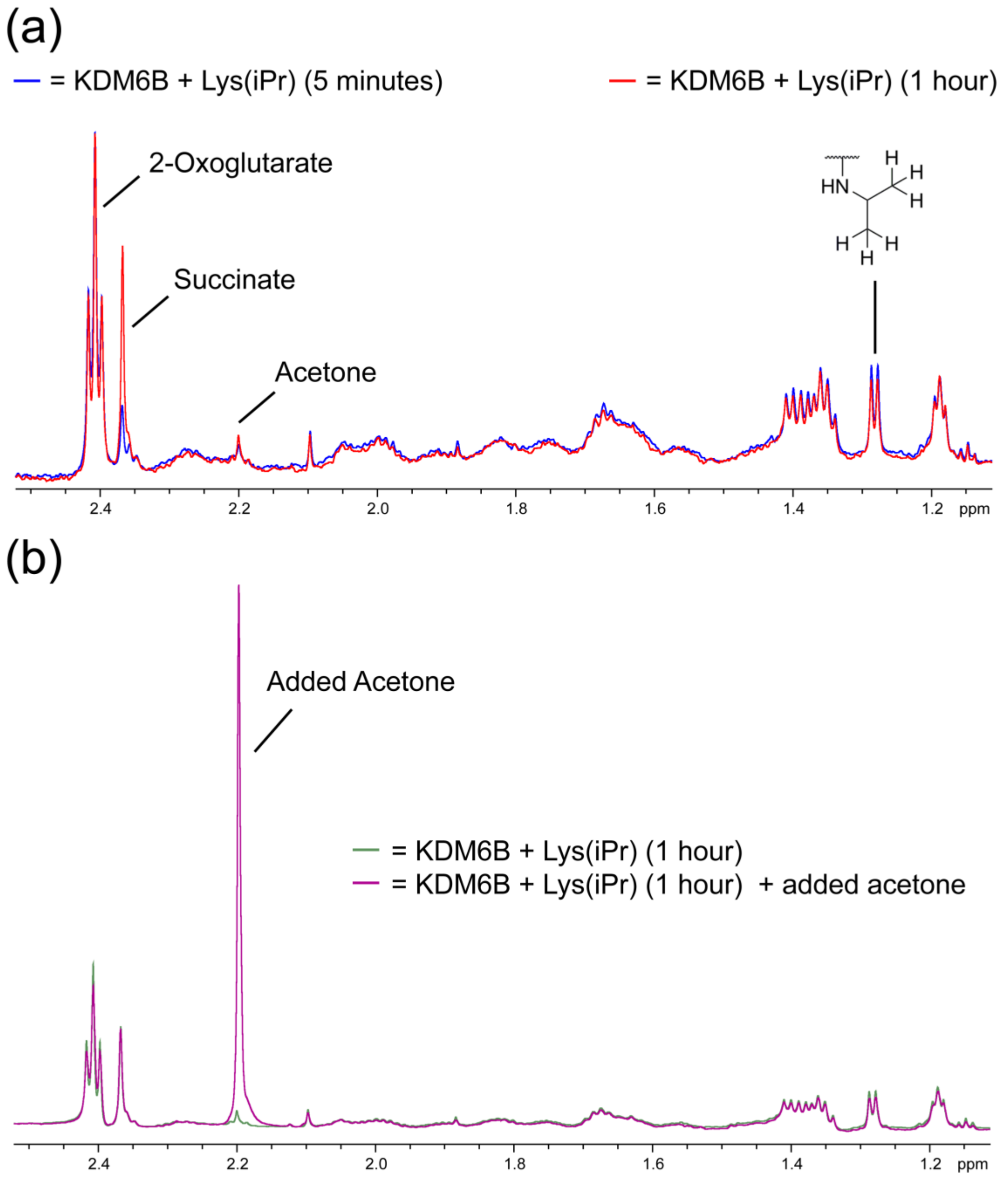

Figure S3. ^{1}H NMR spectra of KDM6B-catalysed de-isopropylation of the Lys(iPr) peptide. (A) ^{1}H NMR spectra of a sample containing KDM6B, the Lys(iPr) peptide, 2-oxoglutarate, ascorbate and ferrous iron after 5 minutes (blue) and one hour (red). Decreased intensity of the doublet resonance at δ_H 1.3 ppm is observed (corresponding to the Lys(iPr) isopropyl protons), with a concomitant small increase in the intensity of the singlet resonance at δ_H 2.2 ppm. Addition of authentic acetone to the sample resulted in an additional increase in the resonance, suggesting a product of de-isopropylation is acetone (B).

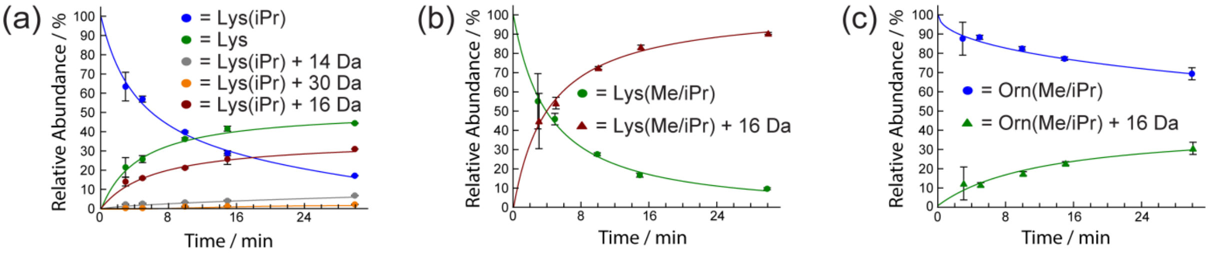

Figure S4. MALDI-MS time-courses of KDM6B-catalysed reactions on histone fragment peptides containing Lys(iPr) (A), Lys(Me/iPr) (B) and Orn(Me/iPr) (C) analogues. Relative abundances of the reactants and products in the mixtures are reported.

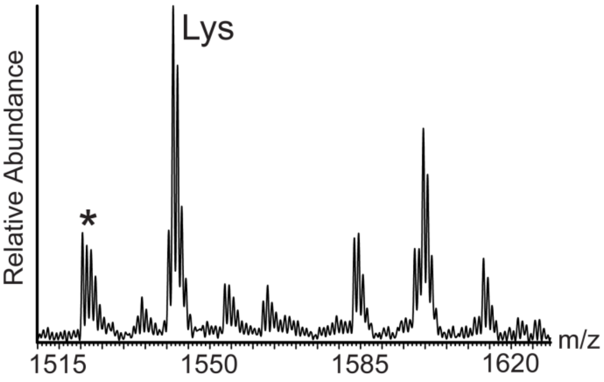

Figure S5. MALDI-MS spectrum of a sample containing KDM6B, the Lys(iPr) peptide, 2-oxoglutarate, ascorbate and ferrous iron after one hour incubation at 25 °C. The emergence of a new peak (asterisked) suggests loss of 63 Da from the Lys(iPr) peptide, which is larger than the mass of the isopropyl group. However, the relatively low intensity of the peak precludes assignment (note: there are likely two species with similar mass, based on the peak shape).

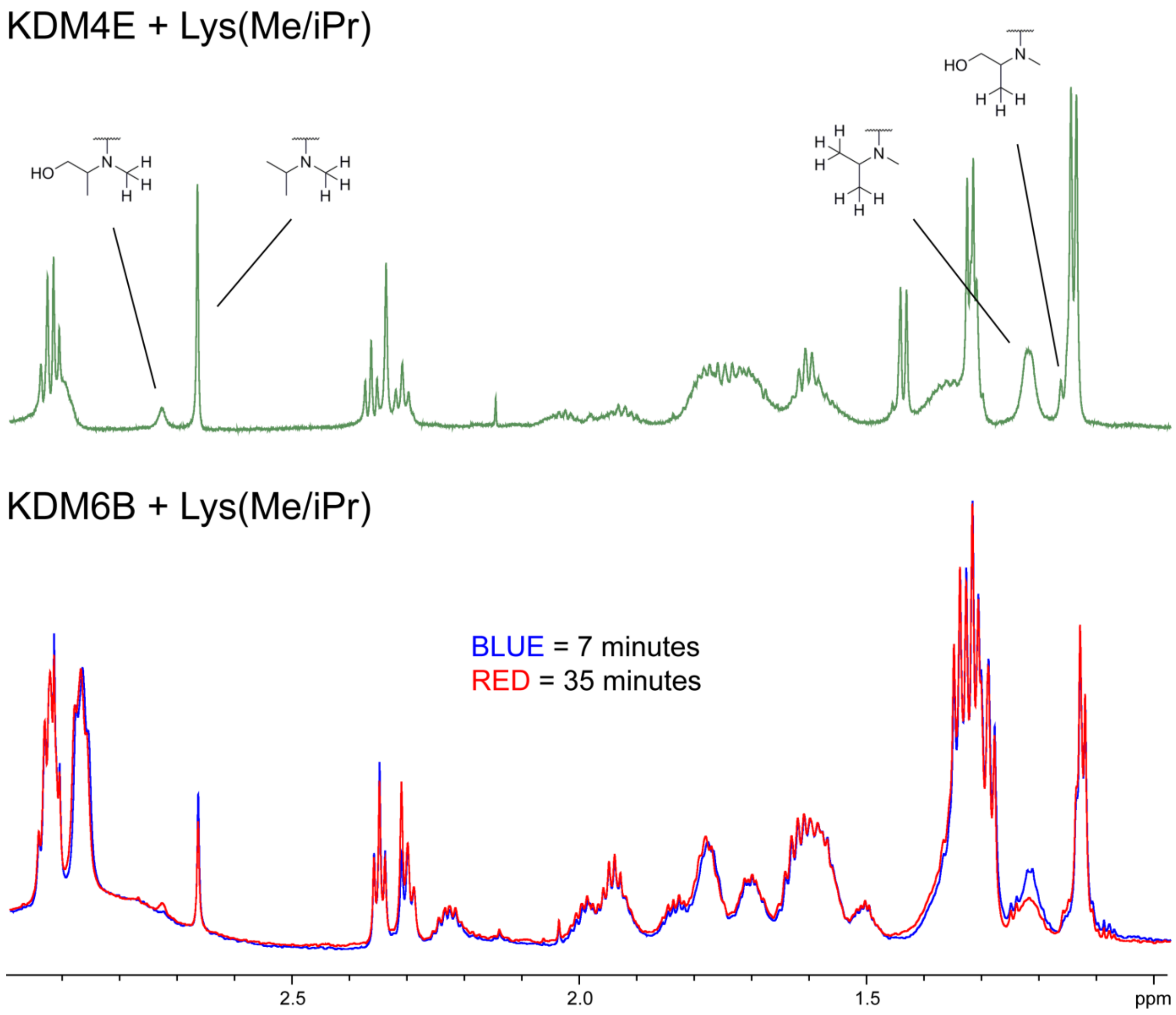

Figure S6. ^{1}H NMR spectra of KDM6B-catalysed oxidation of the Lys(Me/iPr) peptide. ^{1}H NMR spectra of a sample containing KDM6B, Lys(Me/iPr) peptide, 2-oxoglutarate, ascorbate and ferrous iron after 7 minutes (blue) and 35 minutes (red) reveal decreased intensities of the resonances at δ_H 1.2 ppm and δ_H 2.65 ppm, and concomitant emergence of new resonances at δ_H 1.15 ppm and δ_H 2.7 ppm. While the intensities of the new resonances are too low to fully assign, overlay of the spectra with that of an incubation of KDM4E, a histone H3 fragment peptide containing Lys(Me/iPr) at lysine-9, 2-oxoglutarate, ascorbate and ferrous iron (green), suggest the products of both reactions are identical, i.e. that KDM6B catalysis on Lys(Me/iPr) results in hydroxylation on the isopropyl methyl group.

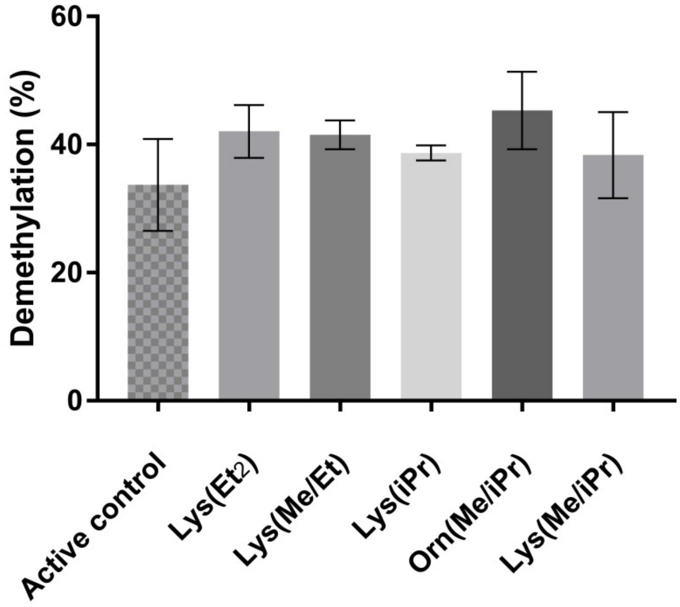

Figure S7. Substrate histone peptide analogues do not inhibit demethylation of the H3K27Me3 21mer substrate peptide. Percentage demethylation was calculated relative to enzyme free control wells after 3 minutes reaction at 25 °C. No reduction in demethylation was observed in samples containing the analogue peptides versus enzyme-only controls. This was confirmed by Dunnett's multiple comparison test, although a low significant difference (P= 0.0497) was detected between active control and the Orn(Me/iPr) peptide-containing samples. Error bars represent standard deviations, where enzyme free control n = 8, enzyme only control n = 6, histone peptide fragment n = 3 technical replicates. One outlier technical replicate was removed from the Lys(iPr) data.

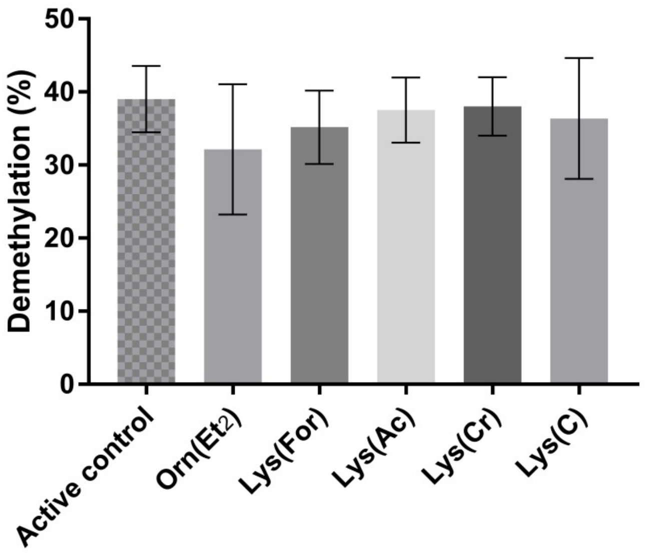

Figure S8. Non-substrate analogue-containing peptides do not inhibit demethylation of H3K27Me3 21mer substrate peptide. No reduction of demethylation was observed in samples containing lysine analogue peptides versus enzyme only controls. This was confirmed by Dunnett's multiple comparison test. Error bars represent standard deviation, where enzyme free control n = 8, enzyme only control n = 6, and histone peptide fragment n = 3 technical replicates.

NMR Spectra of Purified Analogues

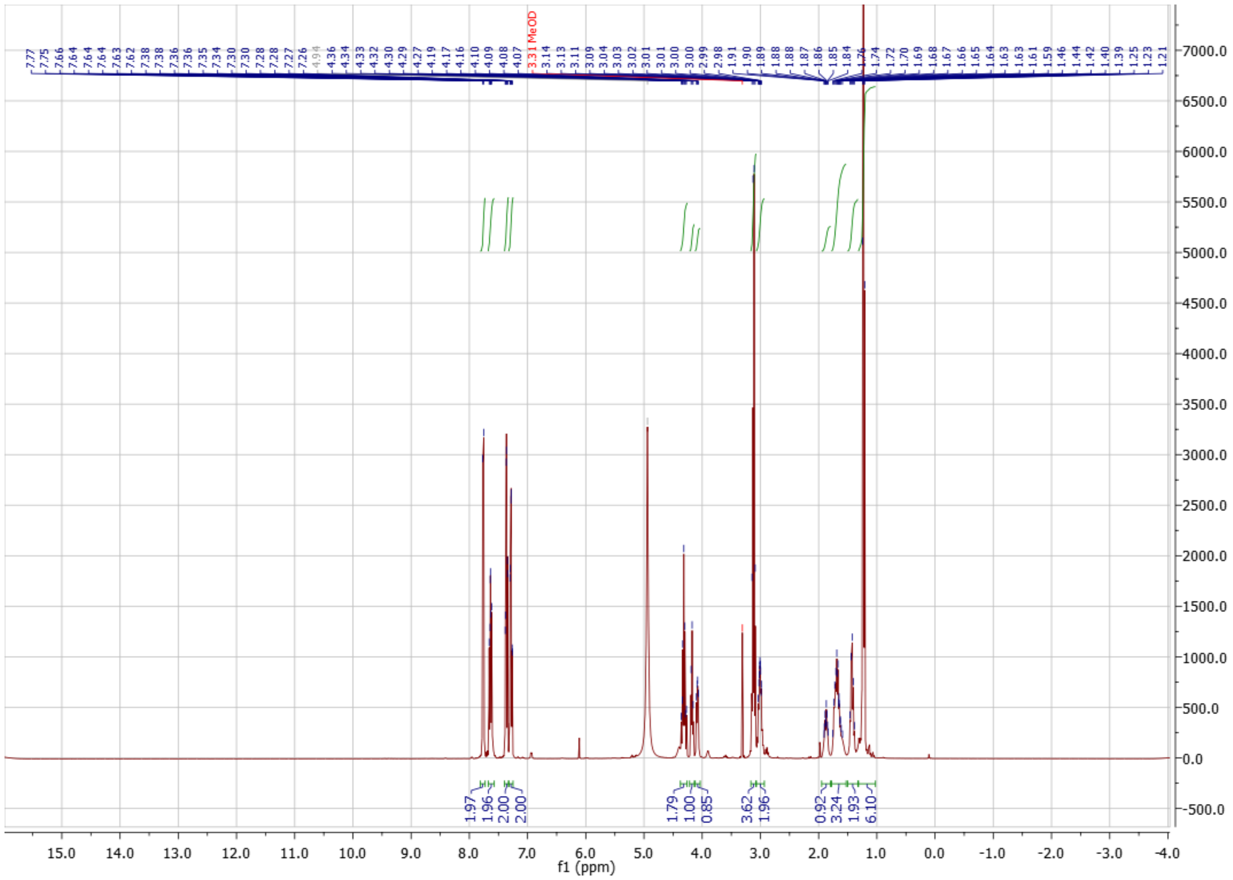

^{1}H NMR spectrum of N^{α}-Fmoc-N^{ε}-diethyl-lysine hydrochloride.

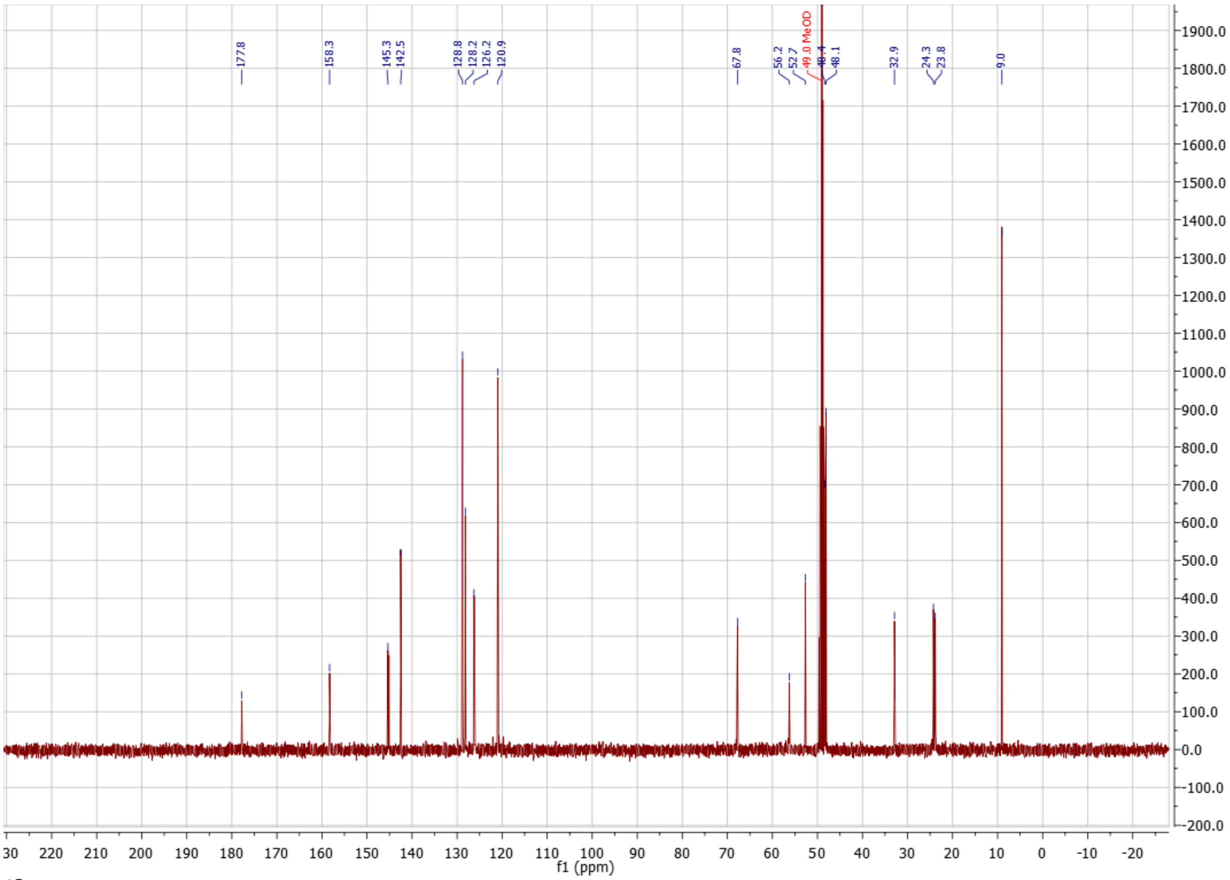

^{13}C NMR spectrum of N^{α}-Fmoc-N^{ε}-diethyl-lysine hydrochloride.

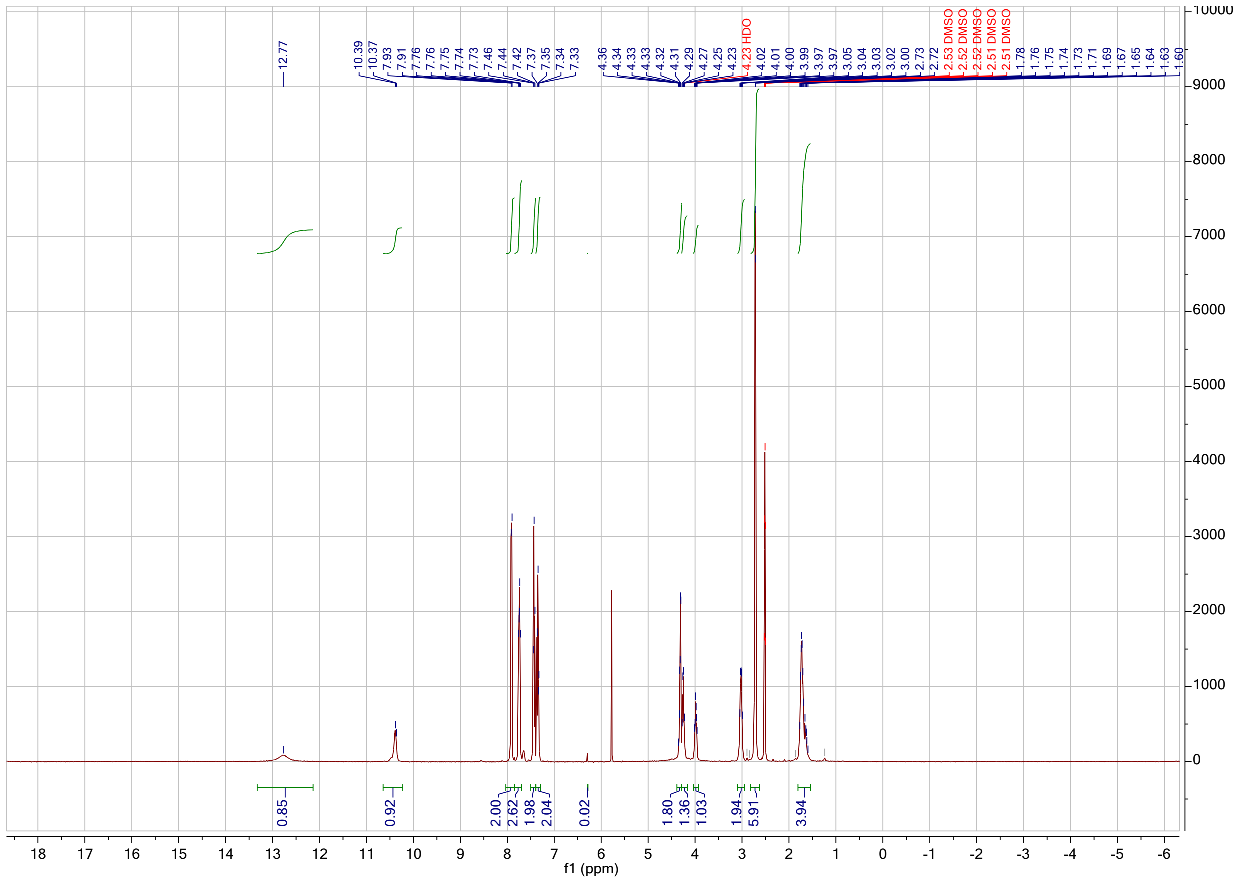

^{1}H NMR spectrum of N^{α}-Fmoc-N^{δ}-dimethyl-ornithine hydrochloride.

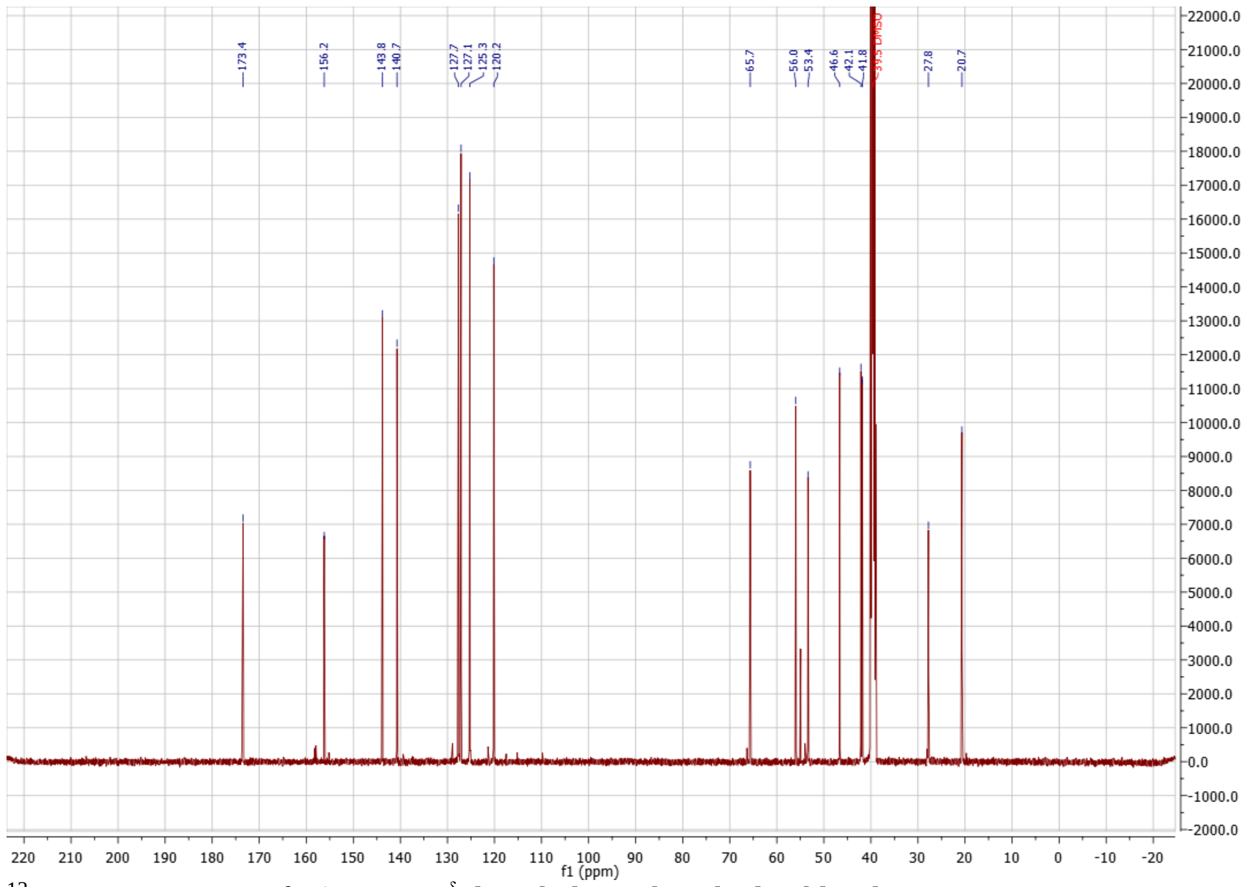

^{13}C NMR spectrum of N^{α}-Fmoc-N^{δ}-dimethyl-ornithine hydrochloride.

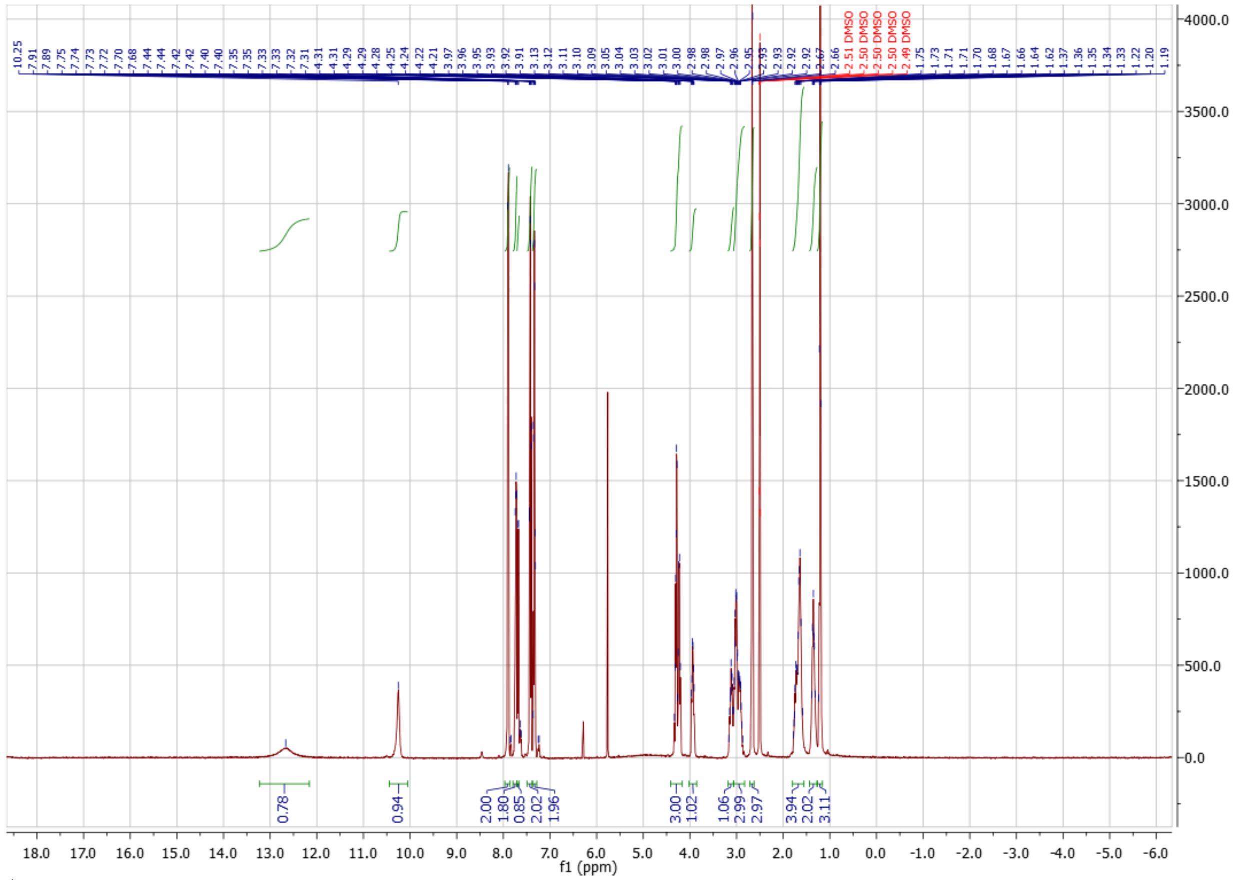

^{1}H NMR spectrum of N^{α}-Fmoc-N^{ε}-ethyl-N^{ε}-methyl-lysine hydrochloride.

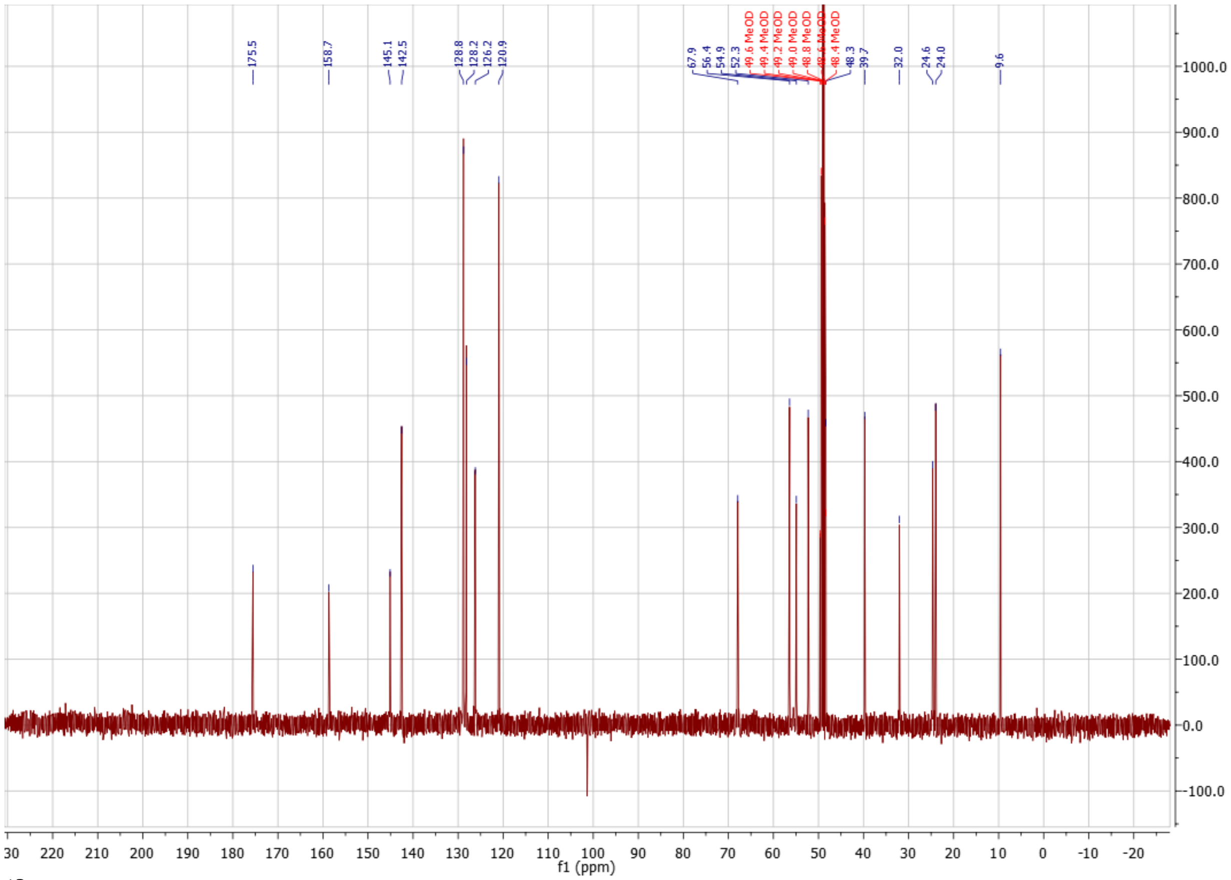

^{13}C NMR spectrum of N^{α}-Fmoc-N^{ε}-ethyl-N^{ε}-methyl-lysine hydrochloride.

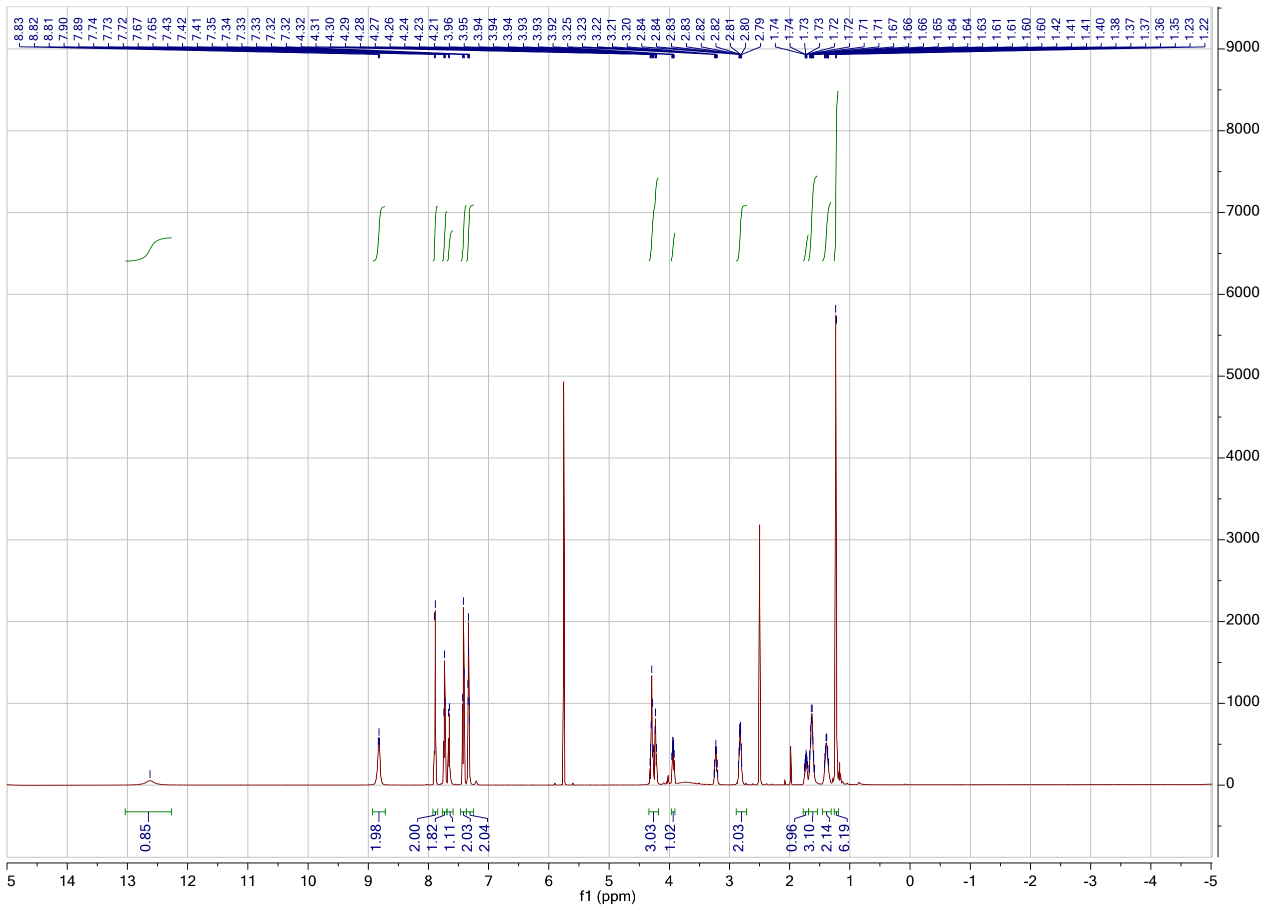

^{1}H NMR spectrum of N^{α}-Fmoc-N^{ε}-isopropyl-lysine hydrochloride.

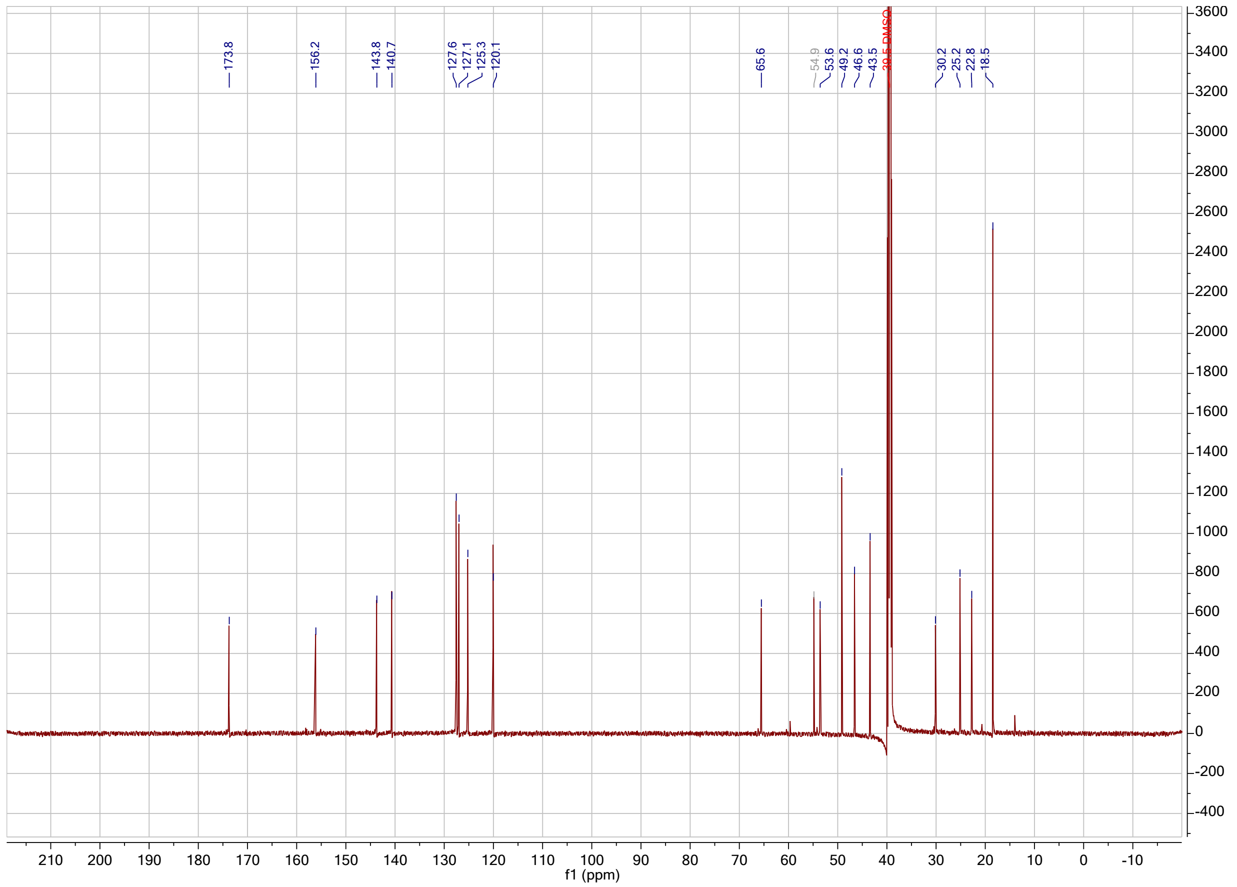

^{13}C NMR spectrum of N^{α}-Fmoc-N^{ε}-isopropyl-lysine hydrochloride.

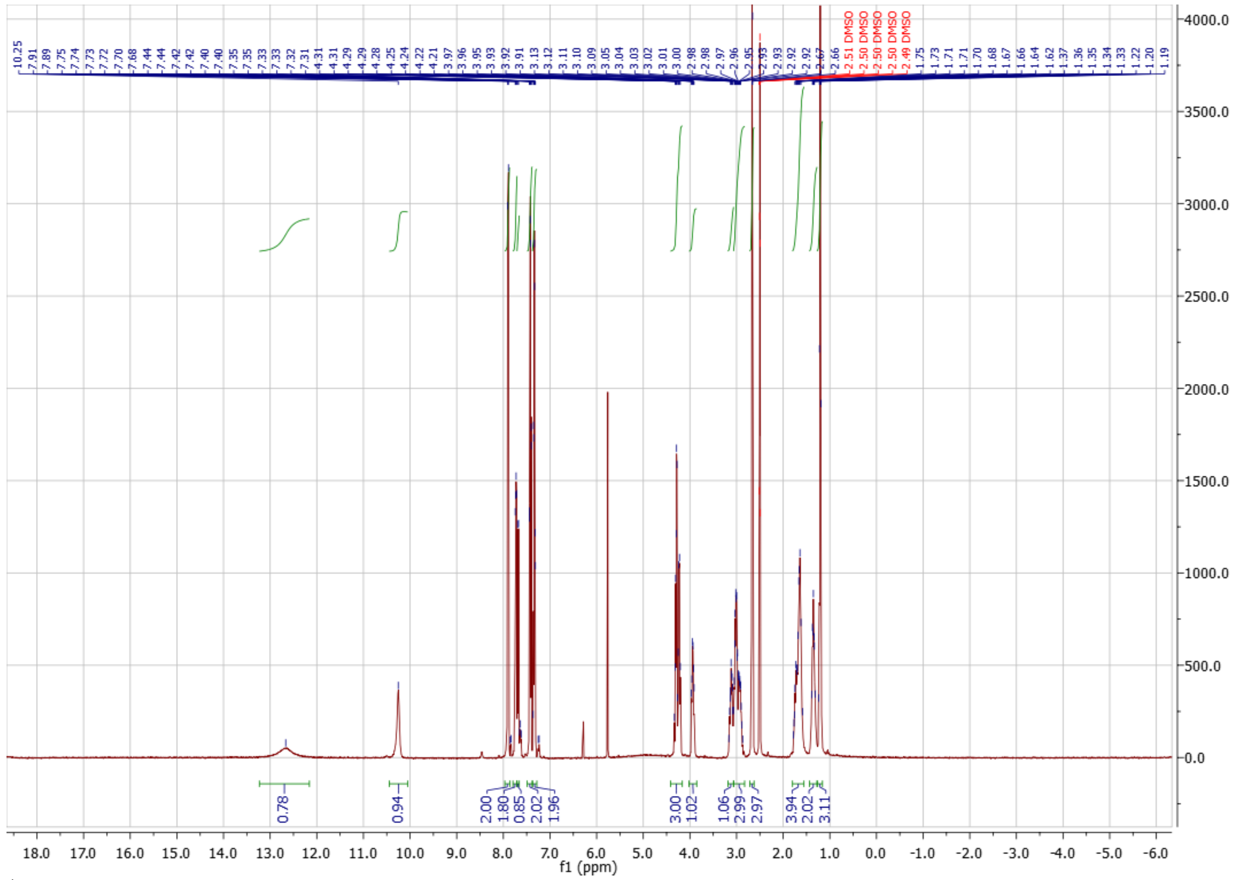

^{1}H NMR spectrum of *N*$^{\alpha}$-Fmoc-*N*$^{\varepsilon}$-isopropyl-*N*$^{\varepsilon}$-methyl-lysine hydrochloride.

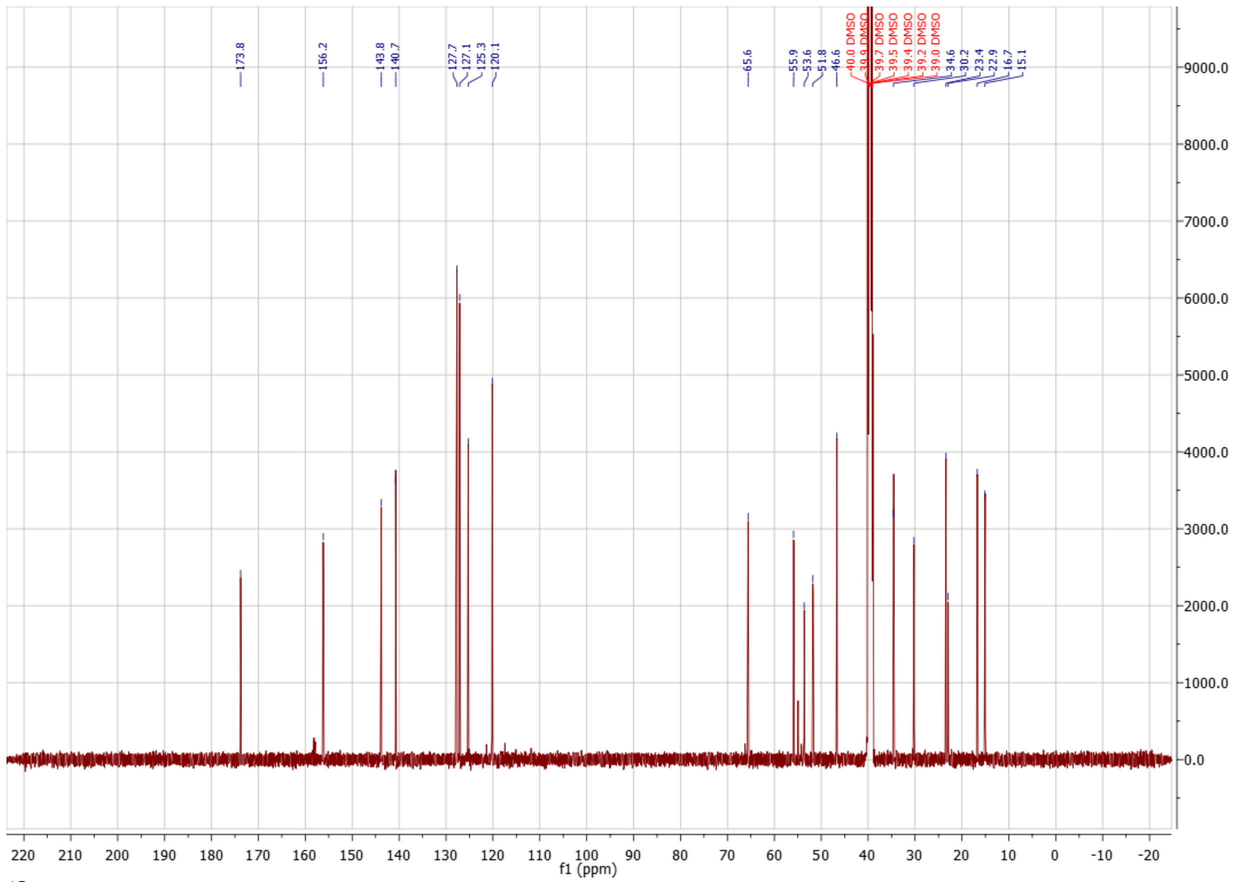

^{13}C NMR spectrum of N^{α}-Fmoc-N^{ε}-isopropyl-N^{ε}-methyl-lysine hydrochloride.

References

1. N. R. Rose, E. C. Woon, A. Tumber, L. J. Walport, R. Chowdhury, X. S. Li, O. N. King, C. Lejeune, S. S. Ng, T. Krojer, M. C. Chan, A. M. Rydzik, R. J. Hopkinson, K. H. Che, M. Daniel, C. Strain-Damerell, C. Gileadi, G. Kochan, I. K. Leung, J. Dunford, K. K. Yeoh, P. J. Ratcliffe, N. Burgess-Brown, F. von Delft, S. Muller, B. Marsden, P. E. Brennan, M. A. McDonough, U. Oppermann, R. J. Klose, C. J. Schofield and A. Kawamura, *J Med Chem*, 2012, **55**, 6639-6643.
2. R. J. Hopkinson, L. J. Walport, M. Münzel, N. R. Rose, T. J. Smart, A. Kawamura, T. D. W. Claridge and C. J. Schofield, *Angewandte Chemie International Edition*, 2013, **52**, 7709-7713.
3. G. W. Langley, A. Brinkø, M. Münzel, L. J. Walport, C. J. Schofield and R. J. Hopkinson, *ACS Chemical Biology*, 2016, **11**, 755-762.
4. R. J. Hopkinson, R. B. Hamed, N. R. Rose, T. D. Claridge and C. J. Schofield, *Chembiochem*, 2010, **11**, 506-510.
